# Effect of alkali metals on physical and spectroscopic properties of cellulose

**DOI:** 10.1038/s41598-023-48850-7

**Published:** 2023-12-08

**Authors:** Ahmed Refaat, Hanan Elhaes, Medhat A. Ibrahim

**Affiliations:** 1 Spectroscopy Department, National Research Centre, 33 El-Bohouth St., Dokki, Giza, 12622 Egypt; 2 Molecular Modeling and Spectroscopy Laboratory, Centre of Excellence for Advanced Science, National Research Centre, 33 El-Bohouth St., Dokki, Giza, 12622 Egypt; 3 Physics Department, Faculty of Women for Arts, Science and Education, Ain Shams University, Cairo, 11757 Egypt

**Keywords:** Electronic structure of atoms and molecules, Computational chemistry, Density functional theory, Biomaterials, Electronic structure

## Abstract

A 3-unit cellulose model molecule was built and optimized using DFT B3LYP/6-31G(d,p). The electronic properties of the optimized structure of cellulose were investigated in terms of total dipole moment (TDM), HOMO–LUMO band gap (ΔE), and molecular electrostatic potential (MESP). Cellulose demonstrated a TDM of 9.106 Debye and ΔE of 7.647 eV. The hydrogen atom of the hydroxyl group of the CH_2_OH group of each cellulose unit was replaced by an alkali metal atom (X) such that the 3-unit cellulose once had 1X atom, then 2X, then 3X atoms, where X = Li, Na or K, both without and with 2, 4 and 6 water molecules (W), respectively, to study also the effect of hydration. Without hydration, the values of TDM decreased for all of the proposed interaction, but increased with hydration, while ΔE decreased in all interactions, confirming that interaction cellulose-alkali metal interaction, especially with hydration, resulted in more reactive structures. Mapping of HOMO–LUMO and MESP indicated significant change in the electron density distribution around cellulose under the effect of interaction with the alkali metals, both with and without hydration. The plots of projected density of states also clearly demonstrated the contribution of each alkali metal as well as water in the molecular orbitals, reflecting their effect on the electronic properties of cellulose and cellulose-alkali metals composites. The theoretical calculations were experimentally verified using FTIR and FT-Raman spectroscopy.

## Introduction

Cellulose is the main and most important component in plants and is, therefore, the most abundant material on earth^1^. As a natural biopolymer, cellulose and its derivatives possess numerous advantageous properties such as low toxicity, biocompatibility and biodegradability^2, 3^. It is composed of unbranched chains of glucose molecules linked via beta 1–4 glycosidic bonds and is organized into organize into fibers via hydrogen bonding^4^. Cellulose is characterized by its high chemical functionality owing to the presence of primary and secondary hydroxyl (OH) groups on its surface^5^. These OH groups can be easily modified with different material, resulting in what is known as cellulose derivatives^6^, and can also undergo, mainly, electrostatic hydrogen bonding with one another resulting in an ordered structure^7, 8^. The intramolecular and intermolecular hydrogen bonds of cellulose chains play an important role in both its mechanical properties and in the adsorption of different materials to cellulose^9, 10^ for different applications such as antifouling^11–14^, biomedical applications^15–18^, coatings^19–23^, and green electronics and energy storage devices^24–30^. Cellulose is also currently making its way in several other advanced applications, such as in optoelectronic devices as a replacement to petrochemical-based polymers owing to its ability to manage light interactions^31–33^.

Alkali metals can be easily ionized due to their low ionization energy and, consequently, they are perfect candidates to functionalize different materials by creating an excess electron system using their valence electrons^34^. Therefore, doping with alkali metals is frequently reported in the literature where several studies guided by theoretical calculations have reported that doping with alkali metal atoms (Li, Na, and K) can tune electronic properties and enhance the electrostatic interactions of different materials such as carbon materials^35–38^, zeolitic imidazolate framework^39^, amino and carboxylic acids^40, 41^, and polyaniline^42^ for a variety of applications.

Aided by different methods and numerous levels of theory, molecular modeling has proven to be a very valuable computational approach to study different systems and classes of materials at the electronic and atomistic levels to investigate their electronic, structural and thermodynamic properties, serving as a guide or a complementary approach to the experimental methods^43, 44^. Using molecular modeling, it is also possible to theoretically compute infrared, Raman and NMR spectra with high accuracy and considerable agreement with the experimental ones^45, 46^. Density functional theory (DFT) using B3LYP-D3BJ function coupled with the 6-31g(d) basis set was used to quantify the stability of cellulose II nanocrystals^47^. Dispersion-corrected DFT was implemented to investigate the phonon properties and thermodynamics of four crystalline cellulose allomorphs^48^. DFT in a plane-wave pseudopotential method was implemented to study the adsorption of acetaminophen, sulfamethoxazole, and *N*,*N*-diethyl-*meta*-toluamide from aqueous solution on nanocellulose composites by electrostatic interactions^49^. B3LYP and OPBE functionals, and TZP basis set DFT—Broken Symmetry calculations were conducted to study the interactions within Nanocellulose/Fe_3_O_4_ hybrid nanocomposites^50^. Various combinations of DFT functionals and basis sets were applied to investigate the crystal structures of the native cellulose Iα and Iβ allomorphs^51^. The adsorption of silver ions on the surface of microspheres of native cellulose and cellulose derivatives was studies using a combination of DFT functionals^52^. DFT calculations with dispersion corrections were performed to thoroughly study Iα and Iβ cellulose each in four different conformations^53^.

The aim of the present work is to study the effect of functionalization with the alkali metals Li, Na and K on the electronic properties of cellulose with and without hydration, using DFT molecular modeling calculations at B3LYP/6-31g(d,p) level, in terms of some important parameters; namely, total dipole moment (TDM), highest occupied molecular orbital-lowest unoccupied molecular orbital (HOMO–LUMO) band gap (ΔE), molecular electrostatic potential (MESP), and projected density of states (PDOS). At the same level, both infrared and Raman spectra were calculated to assess the effect of alkali metals on the vibrational characteristics of cellulose. Experimental verification of the theoretical calculations was carried out using FTIR and FT-Raman spectroscopy.

## Materials and methods

### Materials

Microcrystalline cellulose was purchased from S D Fine-Chem Limited (SDFCL), India.

### Methods

#### Fourier transform infrared spectroscopy

Attenuated total reflection (ATR) FTIR spectrum of microcrystalline cellulose was obtained using Vertex 80 FTIR spectrometer from Bruker Optik GmbH, Germany, equipped with diamond ATR crystal system in the spectral range of 4000–400 cm^−1^ with the resolution of 4 cm^−1^.

#### Raman spectroscopy

Raman spectra were collected on WITec Alpha300 R confocal Raman microscope (Germany), with laser 785 nm and maximum power 20 mW. The spectra were recorded over the range 1800–800 cm^−1^ using an operating spectral resolution of 2.0 cm^−1^ of Raman shift. Spectra were taken with 20s exposure and 30 mW laser power output.

### Molecular modeling

#### Calculations details

All the studied models were subjected to quantum mechanical calculations using GAUSSIAN 09^54^ softcode at Molecular Modeling and Spectroscopy Laboratory, Centre of Excellence for Advanced Science, National Research, Egypt. Geometry optimization was done using DFT at B3LYP/6-31g(d,p)^55–57^ level which was also used to calculate TDM, ΔE, MESP, density of states, and IR and Raman frequencies. PDOS plots were generated using GaussSum^58^.

#### Building model molecules

**Figure 1:**
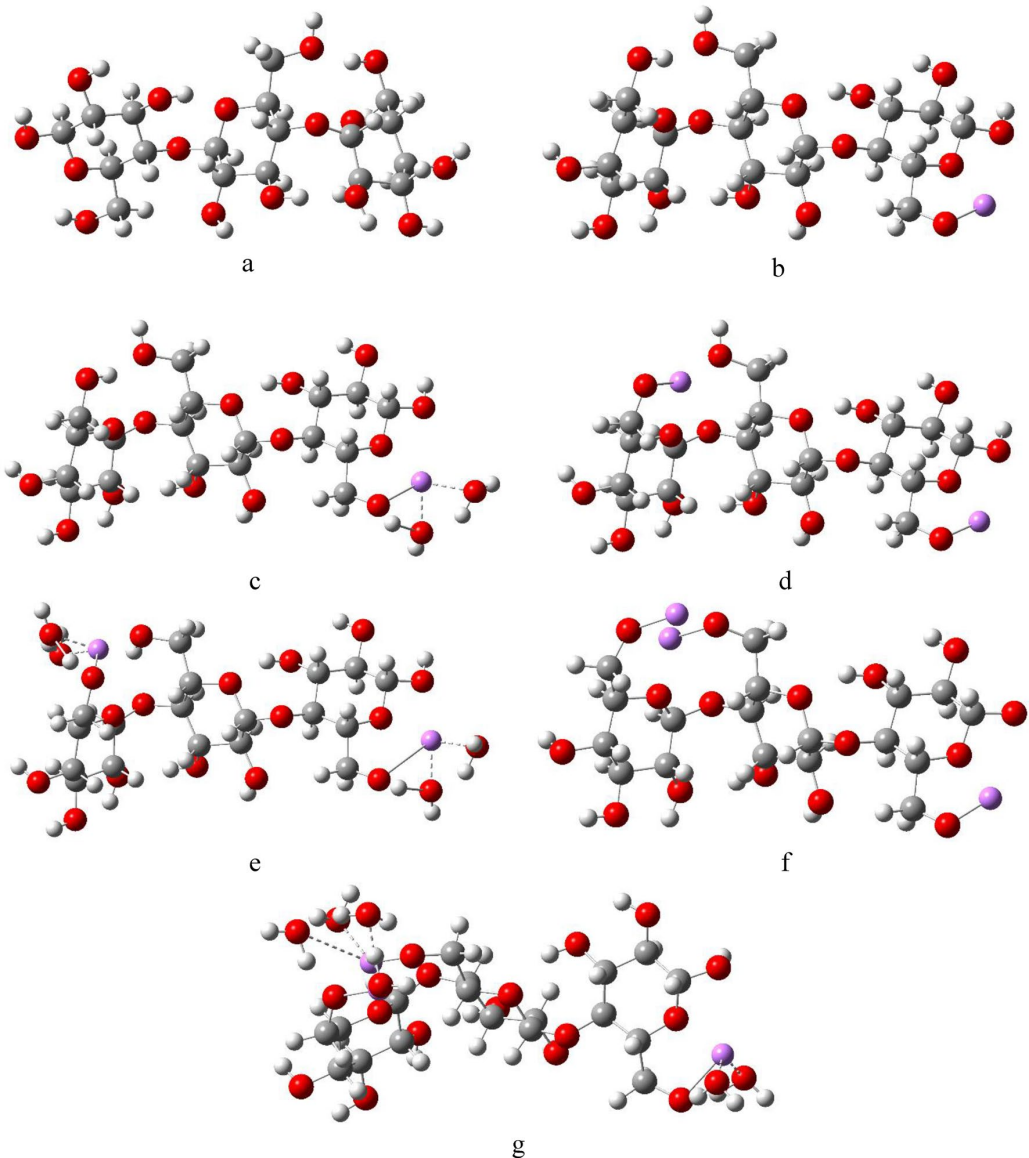
DFT:B3LYP/6-31g(d,p) optimized structures of (**a**) Cellulose; (**b**) Cellulose-1Li; (**c**) Cellulose-1Li2W; (**d**) Cellulose-2Li; (**e**) Cellulose-2Li4W; (**f**) Cellulose-3Li and (**g**) Cellulose-3Li6W.

**Figure 2:**
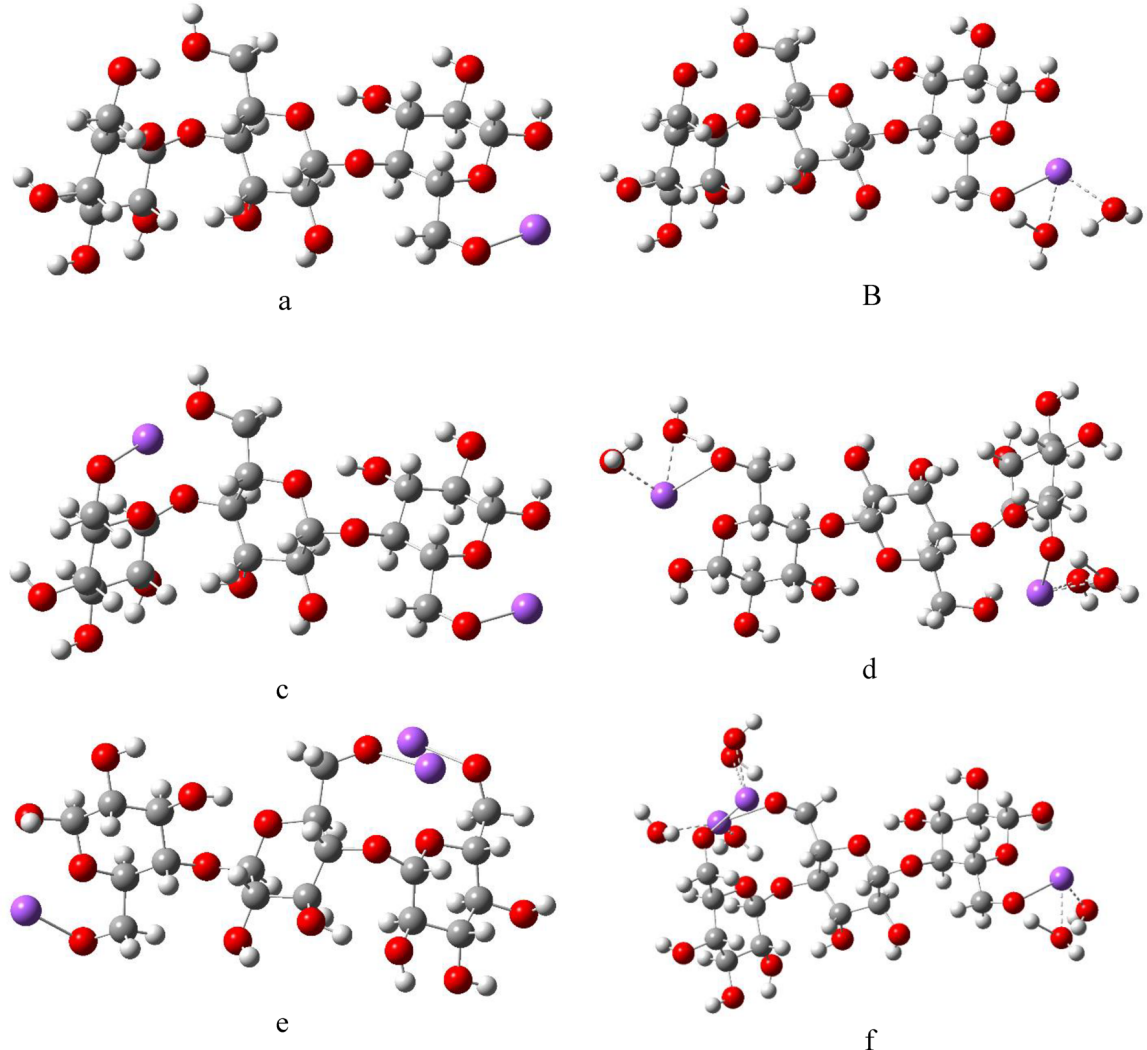
DFT:B3LYP/6-31g(d,p) optimized structures of (**a**) Cellulose-1Na; (**B**) Cellulose-1Na2W; (**c**) Cellulose-2Na; (**d**) Cellulose-2Na4W; (**e**) Cellulose-3Na and (**f**) Cellulose-3Na6W.

**Figure 3:**
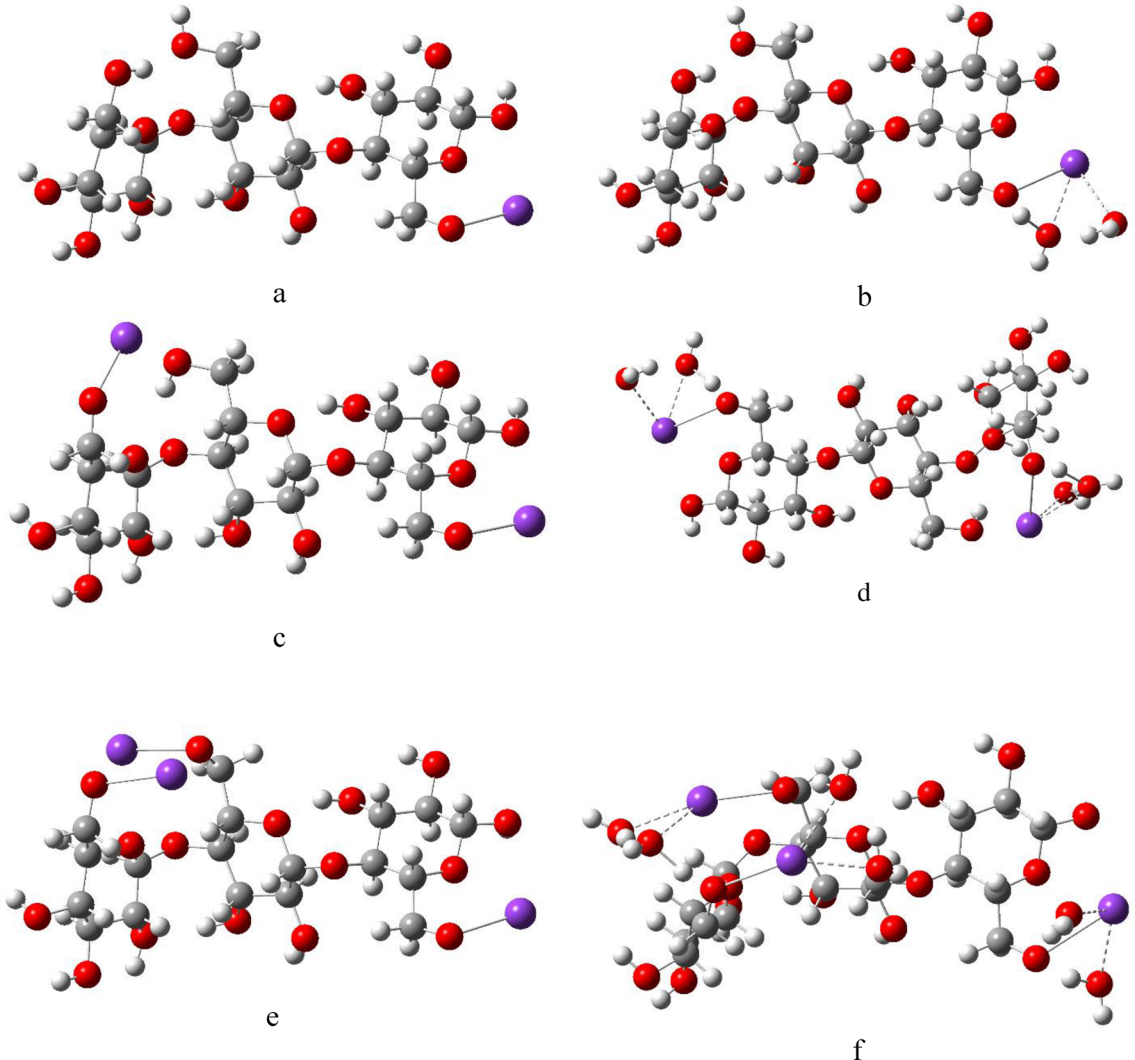
DFT:B3LYP/6-31g(d,p) optimized structures of (**a**) Cellulose-1K; (**b**) Cellulose-1K2W; (**c**) Cellulose-2K; (**d** Cellulose-2K4W; (**e**) Cellulose-3K and (**f**) Cellulose-3K6W.

A 3-unit cellulose model molecule was designed using GaussView 5.0^59^. Each unit is linked to the next via an O-linkage, and contains a CH_2_OH group. The interaction of cellulose with Li, Na or K is proposed to take place by replacing the hydrogen atom of the OH of the CH_2_OH group, and this interaction is to take place once with 1 alkali metal atom, then 2, then 3 atoms. The effect of hydration is also studied by bonding each alkali metal atom to 2 water molecules (W) via hydrogen bonding. This scenario gives a total of 6 interactions to be studied for each alkali metal with cellulose, such that the interaction without hydration is termed Cellulose-X, Cellulose-2X and Cellulose-3X, where X = Li, Na or K, and with hydration is Cellulose-X2W, Cellulose-2X4W and Cellulose-3X6W. The designed molecules were then subjected to geometry optimization, followed by infrared frequency calculation to confirm that the optimized structures are corresponding to true minimum energies with the absence of imaginary frequencies^60^. The optimized structures of cellulose and cellulose-Li, cellulose-Na, and cellulose-K with and without hydration are shown in Figs. 1, 2 and 3, respectively.

### Ethical approval

This work is not applicable for both human and/or animal studies.

## Results and discussions

### Theoretical IR band assignments

**Figure 4:**
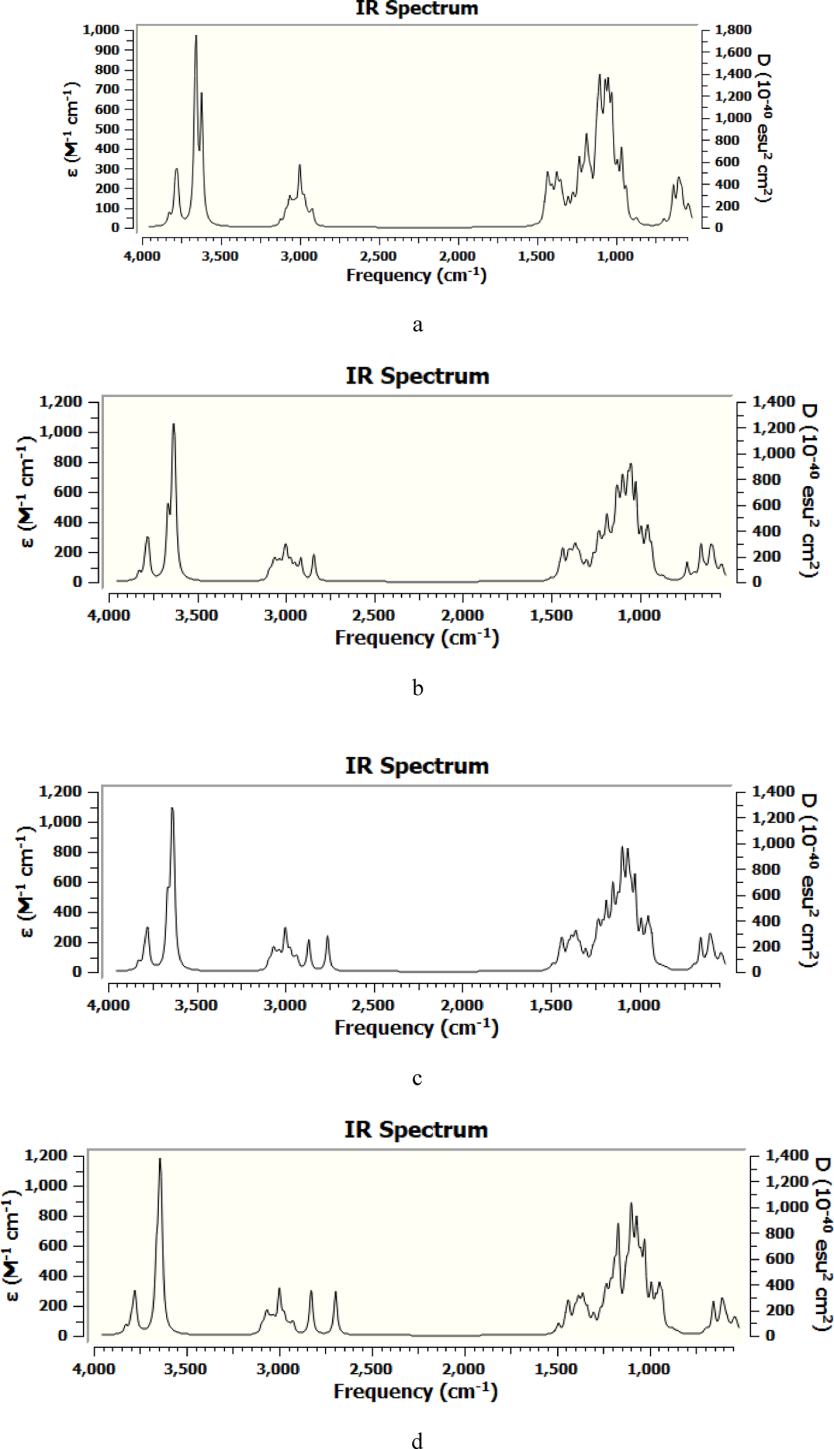
DFT:B3LYP/6-31g(d,p) computed IR spectra of (**a**) Cellulose; (**b**) Cellulose-1Li; (**c**) Cellulose-1Na and (**d**) Cellulose-1K.

Figure 4 demonstrates the DFT:B3LYP/6-31g(d,p) calculated IR spectra of cellulose, cellulose-1Li, cellulose-1Na and cellulose-1K. The rest of the calculated IR spectra can be found as Supplementary Figs. S1–S3. The calculated spectra were visualized and generated using GaussView software and the assignment is provided by the software. To plot the computed IR spectra, the intensities were convoluted with Lorentzian functions of FWHM of 10 cm^−1^ by GaussView. The detailed band assignments of the calculated IR spectra of cellulose, cellulose-1Li, cellulose-1Na and cellulose-1K are shown in Table 1.

**Table 1:** Band assignment of the DFT:B3LYP/6-31g(d,p) calculated IR spectra of cellulose, cellulose-1Li, cellulose-1Na, cellulose-1K.

Cellulose	Cellulose-1Li	Cellulose-1Na	Cellulose-1K	Assignment
3660, 3626	3669–3641	3668, 3645	3665, 3648	Stretching of OH groups of cellulose^61^
3100–2900	3100–2800	3100–2800	3100–2800	CH stretching vibrations^61^
1460–1400	1460–1400	1460–1400	1460–1400	CC stretching, and CH and OH wagging^62^
1378	1370	1376	1376	CC stretching and CH wagging^62, 63^
1326	1326	1328	1327	CH and OH wagging^62^
1257, 1211	1258, 1213	1258, 1215	1258, 1214	C–O–C stretching^63, 64^
1128	1126	1126	1125	C–O stretching^65^
1095	1096	1094	1097	C–O stretching^66^
1078	1077	1073	1074	C–O stretching^67^
1020	1020	1020	1018	C–O stretching^62^
990	984	980	976	C–O stretching^62^
668	666	–	–	C–OH out of plane bending^61, 67^

Correlating the computed IR data together, the prominent and persistent finding was the shift of the OH stretching vibrational bands of cellulose from 3660 and 3626 cm^−1^ to higher wavenumbers as a result of interaction with the alkali metals. This blue shift reflects the interaction between the metal's atom and the OH group of the CH_2_OH moiety of celluose, resulting in the exhaustion of hydroxyl groups and consequent reduction in the intramolecular and intermolecular hydrogen bonds^68, 69^. Regarding cellulose-Li structures, the Li–O bond stretching vibrations were found at 470–450 cm^−1^^70–73^ and the intensity of such vibrations was found to increase with increasing the number of lithium atoms interacting with cellulose. The specific vibration of Na–O bond was found at 440–430 cm^−1^ in cellulose-Na structures^74^. Finally, the band corresponding to K–O bond vibration in the three cellulose-K structures was found at 350–340 cm^−1^ spectral region without significant change in the intensity^75^.

### Experimental FTIR spectroscopy

**Figure 5:**
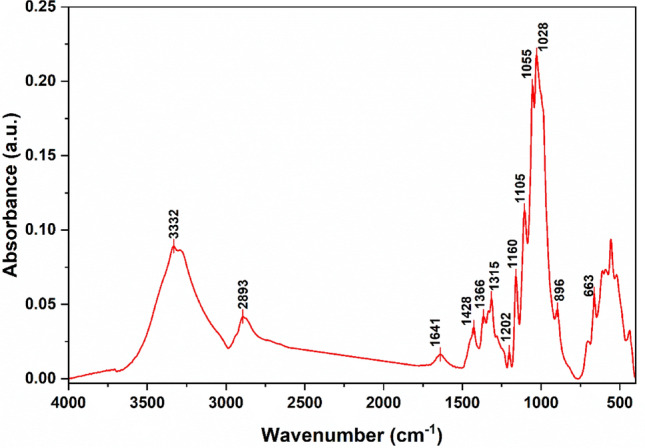
ATR-FTIR spectrum of Cellulose.

The ATR-FTIR spectrum of microcrystalline cellulose is demonstrated in Fig. 5. The band assignment is presented as reported in the literature^61, 63, 65–67^. The band centered at 3332 cm^−1^ corresponding to stretching of OH groups of cellulose and the one at 2893 cm^−1^ is corresponding to CH stretching vibrations. The band at 1641 cm^−1^ is ascribed to –OH bending vibration of absorbed water. The band attributed to symmetric bending of CH_2_ is located at 1428 cm^−1^, while the bands at 1366 and 1315 cm^−1^ are attributed to tertiary CH bending and symmetric wagging of CH_2_, respectively. The symmetric COH in-plane bending vibration is centered at 1202 cm^−1^. The band at 1160 cm^−1^ is related to symmetric stretching of COC of the β-glycosidic linkage, and the band at 1105 cm^−1^ is related to in-plane ring stretching. The bands at 1055 and 1028 cm^−1^ arise from CO stretching and CO asymmetric deformation, respectively. At 896 cm^−1^ appears the asymmetric stretching of COC of the β-glycosidic bond. Finally, the band at 663 cm^−1^ is attributed to out-of-plane bending of COH.

### Theoretical Raman band assignments

A Raman spectrum gives very useful information about the molecular structure and chemical composition, guiding for the identification of material by characterizing its vibrational modes^76^. Obtaining Raman spectra using DFT calculations is deemed very useful in offering valuable information that could be difficult to obtain experimentally, and are free of the possible instrumental contributions, as well as offering the advantage of overcoming preparation problems or unknown phase content^76, 77^.

**Figure 6:**
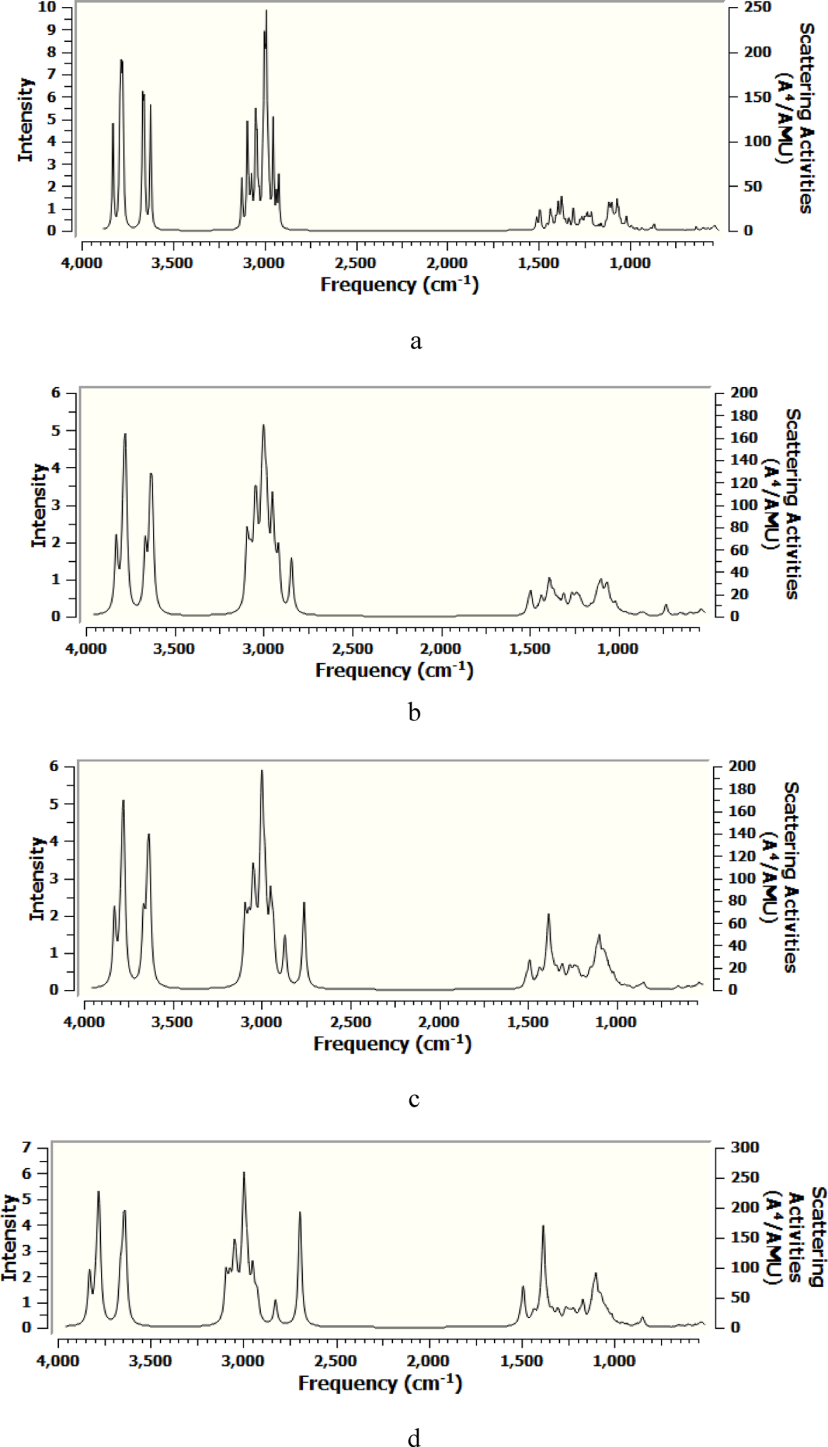
DFT:B3LYP/6-31g(d,p) computed Raman shifts of (**a**) Cellulose; (**b**) Cellulose-1Li; (**c**) Cellulose-1Na and (**d**) Cellulose-1K.

The DFT:B3LYP/6-31g(d,p) calculated Raman shifts of cellulose, cellulose-1Li, cellulose-1Na and cellulose-1K are shown in Fig. 6. The rest of the calculated Raman shifts can be found as Supplementary Figs. S4–S6. The calculated spectra and band assignments were generated provided by GaussView software. The computed intensities were scaled with FWHM of 10 cm^−1^. The band assignments of the calculated Raman shifts of cellulose, cellulose-1Li, cellulose-1Na and cellulose-1K are shown in Table 2.

**Table 2:** Band assignment of the DFT:B3LYP/6-31g(d,p) calculated Raman shifts of cellulose, cellulose-1Li, cellulose-1Na, cellulose-1K.

Cellulose	Cellulose-1Li	Cellulose-1Na	Cellulose-1K	Assignment
3669, 3626	3669, 3641	3668, 3645	3665, 3648	Stretching of OH groups of cellulose^78, 79^
3100–2900	3100–2800	3100–2800	3100–2800	CH stretching vibrations^78–80^
1460–1400	1460–1400	1460–1400	1460–1400	CH_2_ bending^78, 80^
1332	1336	1333	1331	C–C–H, C–O–H, and O–C–H bending^79, 81^
1259	1289, 1265	1289, 1266	1288, 1267	HCC and HCO bending and CH_2_ twisting^79, 81^
1125	1126	1126	1127	C–C Stretching^79, 81^
1098	1096	1104	1105	C–C and C–O Stretching, and COC glycosidic asymmetric stretching^78, 81^
1046	1045	1045	1045	C–C and C–O Stretching^78, 79, 81^
896	887	880	879	Glucose ring deformation and COC in-plane symmetric stretching^78, 81^

As shown in Table 2 and Fig. 6, the DFT-calculated Raman shifts demonstrated a similar behavior to that of IR, such that the noticeable difference between the Raman shifts of cellulose, and cellulose-Li, cellulose-Na and cellulose-K was in the Raman peaks of the stretching of OH groups of cellulose. The lower-wavenumber OH peak (3626 cm^−1^) shifted towards higher wavenumbers in cellulose-Li, cellulose-Na and cellulose-K, owing to the change in the O–H chemical bond upon replacement of the H atom with the alkali metal atom with the consequent changes in bond force and distance, as well as charge density^82, 83^. The higher-wavenumber OH peak (3669 cm^−1^) demonstrated significant decrease in its intensity owing to the consumption of OH group with the replacement of H atom by alkali metal atom^84^.

### Experimental FT-Raman spectroscopy

**Figure 7:**
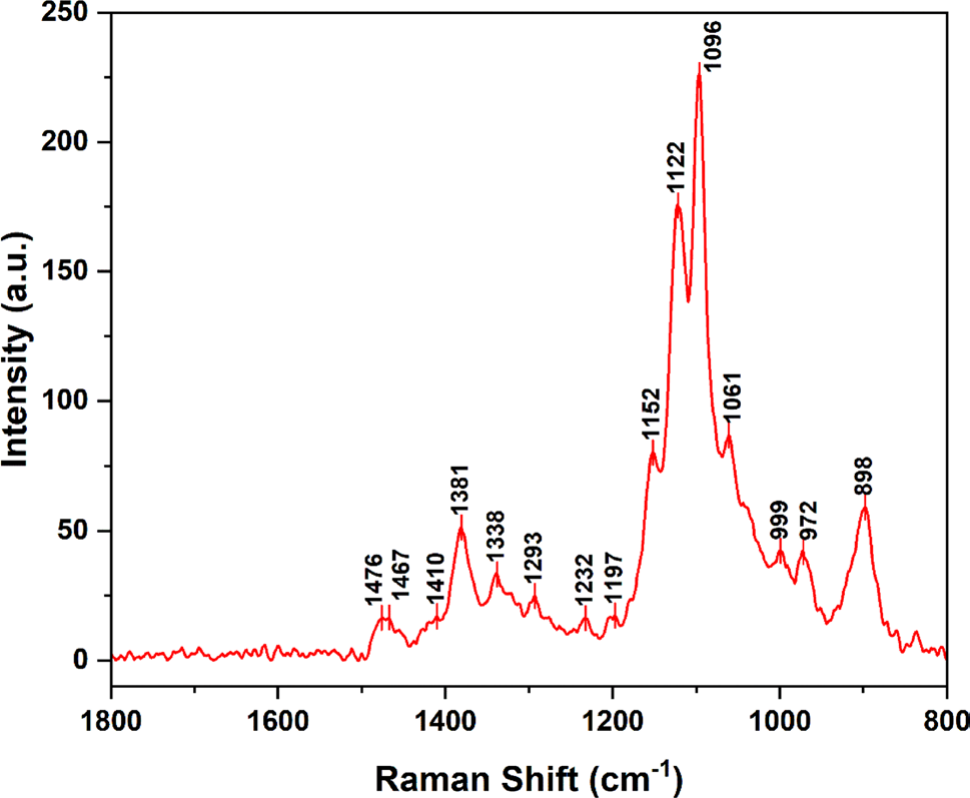
FT-Raman shifts of cellulose.

The Raman shifts spectrum of microcrystalline cellulose is shown in Fig. 7. The characteristic Raman frequencies of cellulose are assigned as reported in the literature^78–81^. The bands at 1476, 1467 and 1410 cm^−1^ are attributed to CH_2_ bending. The bands at 1381 and 1338 cm^−1^ are attributed to C–C–H, C–O–H, and O–C–H bending vibrations. The band at 1293 cm^−1^ is corresponding to HCC and HCO bending and CH_2_ twisting. At 1232 cm^−1^ is centered the band ascribed to COH out-of-plane bending, which the band at 1197 cm^−1^ can be ascribed to COH and CCH bending vibrations. The band at 1152 cm^−1^ is related to asymmetric CC ring stretching. The 1122 cm^−1^ band is attributed to C–C stretching, while the band at 1096 cm^−1^ is attributed to C–C and C–O stretching, and COC glycosidic asymmetric stretching. The band at 1061 cm^−1^ represents C–C and C–O stretching, while the bands at 999 and 972 cm^−1^ represent C–C and C–O stretching, and CH_2_ rocking. The last band at 898 cm^−1^ COC in-plane symmetric stretching.

### Molecular electrostatic potential mapping

**Figure 8:**
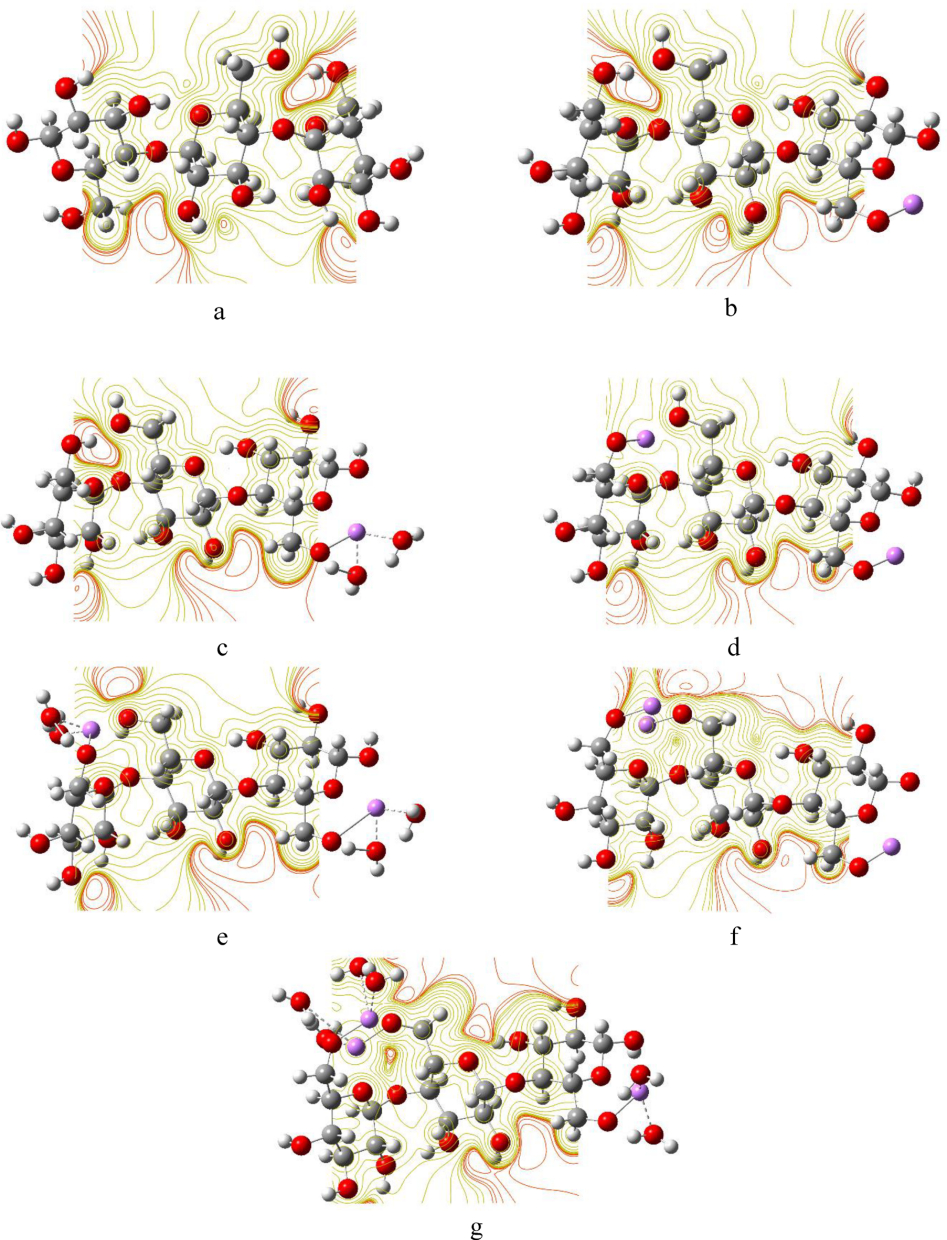
DFT:B3LYP/6-31g(d,p) calculated MESP maps of (**a**) Cellulose; (**b**) Cellulose-1Li; (**c**) Cellulose-1Li2W; (**d**) Cellulose-2Li; (**e**) Cellulose-2Li4W; (**f**) Cellulose-3Li and (**g**) Cellulose-3Li6W.

**Figure 9:**
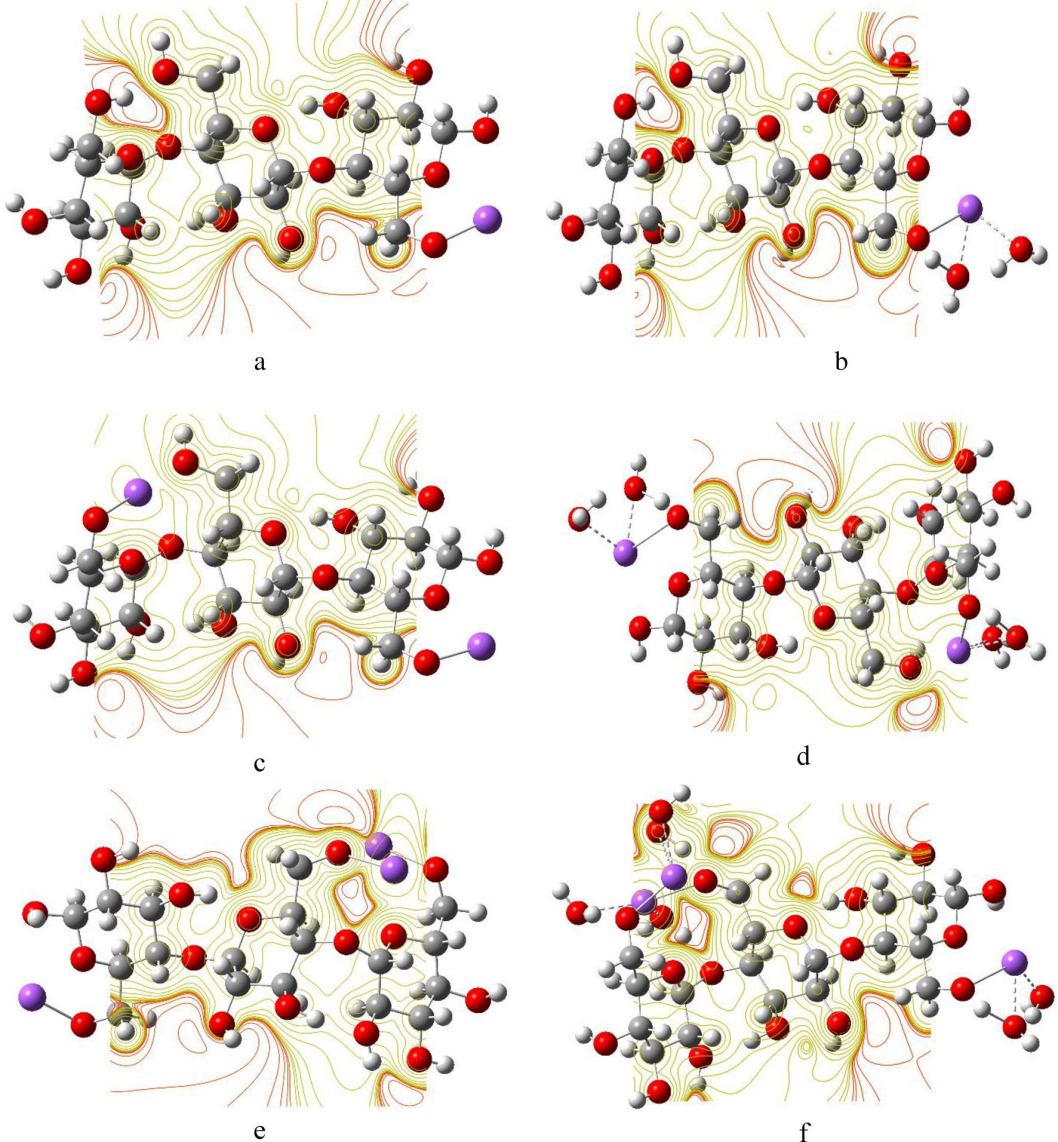
DFT:B3LYP/6-31g(d,p) calculated MESP of (**a**) Cellulose-1Na; (**b**) Cellulose-1Na2W; (**c**) Cellulose-2Na; (**d**) Cellulose-2Na4W; (**e**) Cellulose-3Na and (**f**) Cellulose-3Na6W.

**Figure 10:**
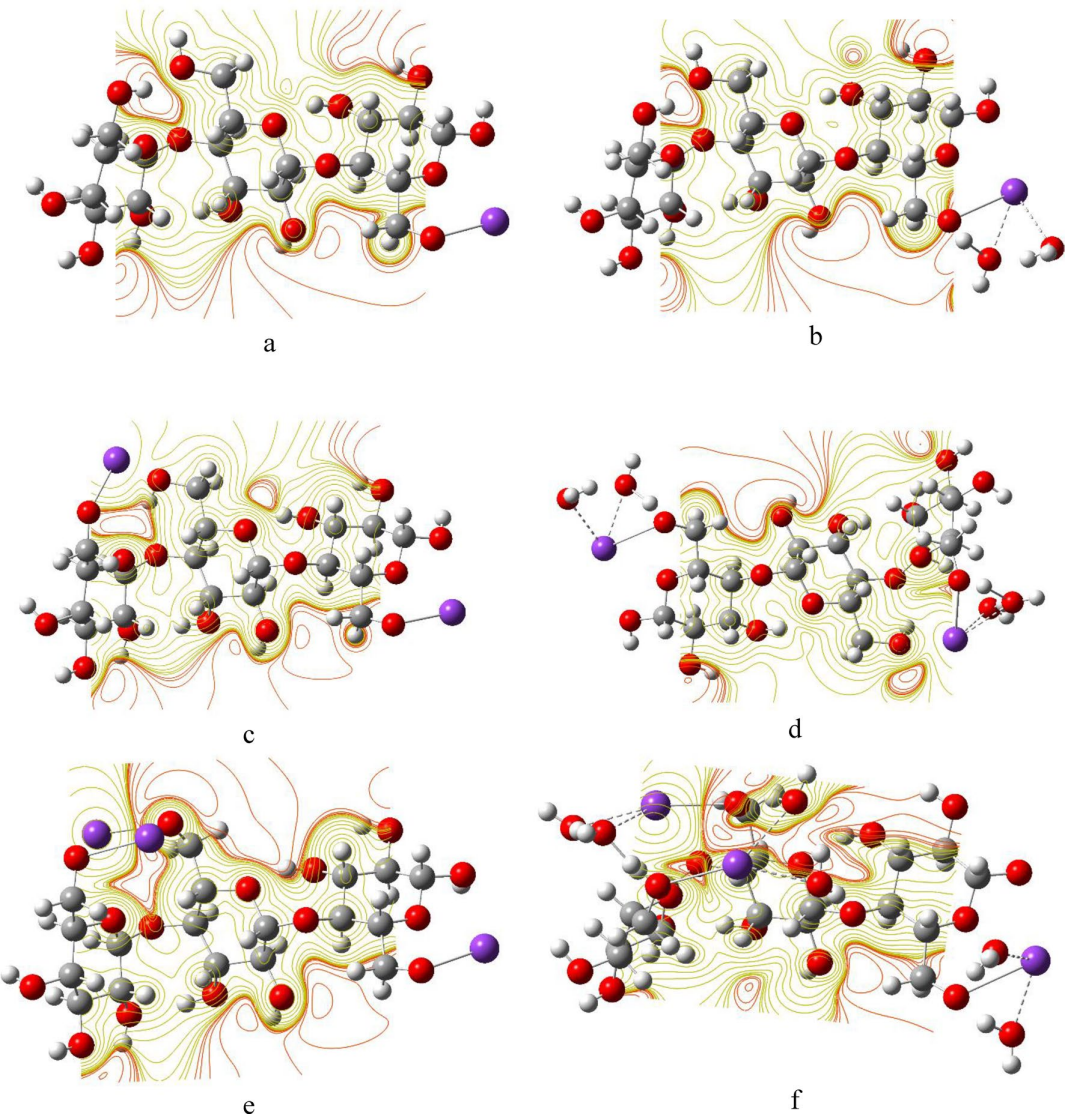
DFT:B3LYP/6-31g(d,p) calculated MESP maps of (**a**) Cellulose-1K; (**b**) Cellulose-1K2W; (**c**) Cellulose-2K; (**d**) Cellulose-2K4W; (**e**) Cellulose-3K and (**f**) Cellulose-3K6W.

The MESP map is a very useful color-coded three-dimensional map that is often used to predict the electron density distribution and to identify the possible sites of electrophilic and nucleophilic attacks^85^. The colors in an MESP map of a given molecule are arranged in a representative order from highest to lowest electron density as red > orange > yellow > green > blue, such that the red color indicates the highest electron density, thus lowest electrostatic potential, while blue represents the highest electrostatic potential^86^. Figures 8, 9 and 10 show the MESP maps of cellulose and cellulose-Li, cellulose-Na, and cellulose-K, respectively. As shown in Fig. 8a, the MESP map of cellulose indicated that the sites of the higher electron density are around the hydroxyl groups of cellulose. In Fig. 8b–g, upon interaction with Li, there was significant change in the MESP maps introducing sites ready for attacked by nucleophiles, with the sites of the higher electron density still being around the hydroxyl groups of cellulose. Similar behavior is demonstrated in the MESP maps of cellulose-Na and cellulose-K as shown in Figs. 9 and 10, respectively.

### Total dipole moment and HOMO/LUMO band gap energy

The TDM is another important physical property of a molecule, since the dipole moments show high sensitivity even to small errors, thus considered an effective check for the efficiency of calculations, and in describing electron density distribution^87^. TDM is also an important descriptor of the reactivity and the effects of impurity atoms on the system, and it has been reported in several studies that it is closely related to reactivity, such that higher TDM reflects higher reactivity^86–88^. In addition, ΔE which is the difference between HOMO and LUMO is also considered an efficient indicator of the reactivity and chemical stability of the molecule^86, 88^.

The computed TDM and ΔE values cellulose and cellulose-Li, cellulose-Na and cellulose-K are demonstrated in Tables 3, 4 and 5, respectively. Cellulose demonstrated a TDM of 9.106 Debye and ΔE of 7.647 eV. The interaction of cellulose with lithium without hydration resulted in decrease in the values of both TDM and ΔE, while with hydration there was increase in TDM and decrease in ΔE for cellulose-1Li2W, cellulose-2Li4W and cellulose-3Li6W, which means that interaction in the presence of hydration resulted in more reactive structures than without hydration.

**Table 3:** DFT:B3LYP/6-31g(d,p) calculated TDM in Debye and ΔE in eV of cellulose, cellulose-1Li, cellulose-1Li2W, cellulose-2Li, cellulose-2Li4W, cellulose-3Li, and cellulose-3Li6W.

Structure	TDM (Debye)	ΔE (eV)
Cellulose	9.106	7.647
Cellulose 1Li	7.697	4.515
Cellulose 1Li 2W	11.806	6.753
Cellulose 2Li	8.460	4.208
Cellulose 2Li 4W	10.277	6.091
Cellulose 3Li	8.083	4.085
Cellulose 3Li 6W	9.893	5.424

Significant values are in [bold].

**Table 4:** DFT:B3LYP/6-31g(d,p) calculated TDM in Debye and ΔE in eV of cellulose, cellulose-1Na, cellulose-1Na2W, cellulose-2Na, cellulose-2Na4W, cellulose-3Na, and cellulose-3Na6W.

Structure	TDM (Debye)	ΔE (eV)
Cellulose	9.106	7.647
Cellulose 1Na	5.756	3.300
Cellulose 1Na 2W	10.857	5.839
Cellulose 2Na	9.826	3.120
Cellulose 2Na 4W	10.400	5.343
Cellulose 3Na	6.321	2.946
Cellulose 3Na 6W	9.741	5.180

Significant values are in [bold].

**Table 5:** DFT:B3LYP/6-31g(d,p) calculated TDM in Debye and ΔE in eV of cellulose, cellulose-1K, cellulose-1K2W, cellulose-2K, cellulose-2K4W, cellulose-3K, and cellulose-3K6W.

Structure	TDM (Debye)	ΔE (eV)
Cellulose	9.106	7.647
Cellulose 1K	4.324	2.719
Cellulose 1K 2W	10.218	5.265
Cellulose 2K	7.940	2.505
Cellulose 2K 4W	10.731	4.750
Cellulose 3K	4.707	2.617
Cellulose 3K 6W	5.899	4.352

Significant values are in [bold].

Regarding cellulose-Na interactions, again without hydration there was decrease in the values of both TDM and ΔE, while with hydration there was increase the values of TDM with simultaneous decrease in ΔE, which again confirms that hydration resulted in more reactive structures.

Cellulose-K interactions showed similar behavior to that of cellulose-Li and cellulose-Na, where in the absence of hydration, the TDM decreased while it increased with hydration, except for cellulose-3K6W. ΔE decreased both without and with hydration, implying that cellulose-K interactions had an enhancing effect on the ΔE.

**Figure 11:**
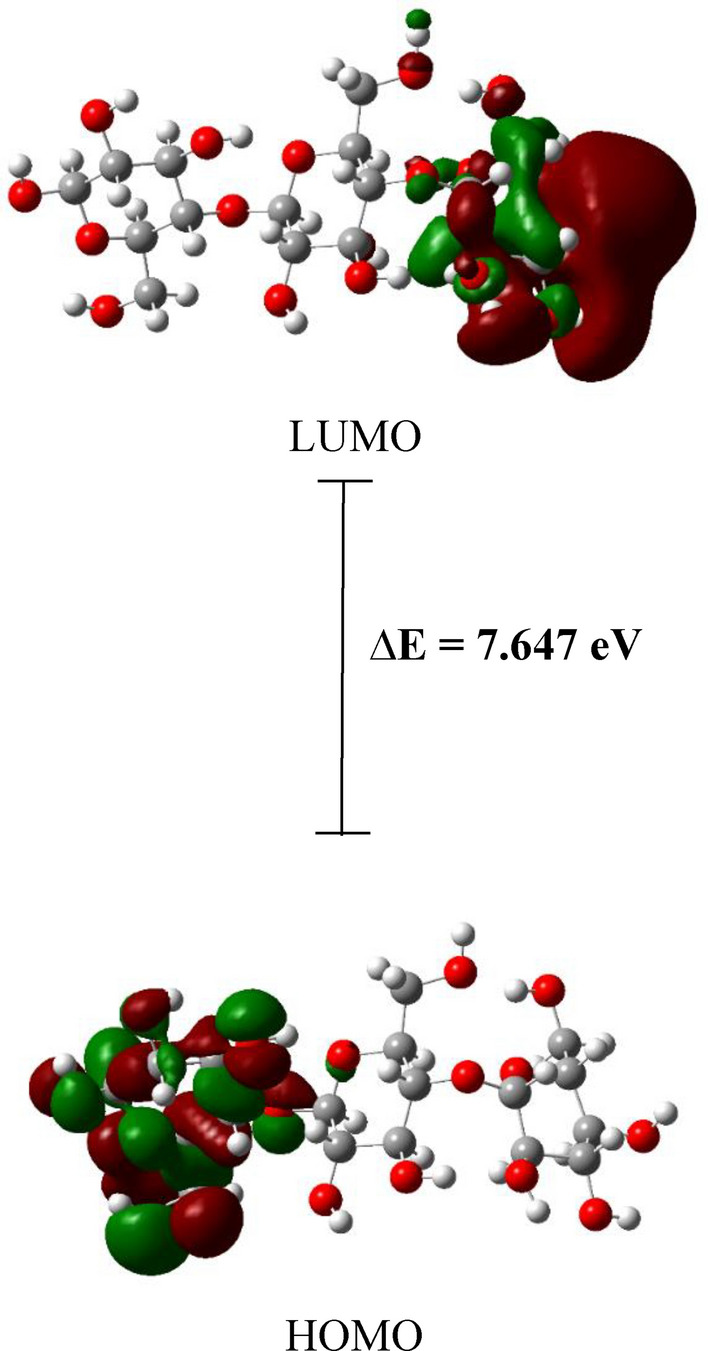
DFT:B3LYP/6-31g(d,p) calculated HOMO/LUMO orbitals of Cellulose.

Plotting the HOMO–LUMO molecular orbitals distribution is also a beneficial approach in visualizing and predicting the reactive sites of the molecules, and to identify the effect of a given interaction on the distribution of the HOMO, as an electron donor, and LUMO, as an electron acceptor, thus identifying sites of nucleophilic and electrophilic attack during bonding interactions^89^. The HOMO–LUMO molecular orbitals distribution of cellulose is shown in Fig. 11, which indicated that both HOMO and LUMO sites are located on the terminal units of the cellulose molecule.

**Figure 12:**
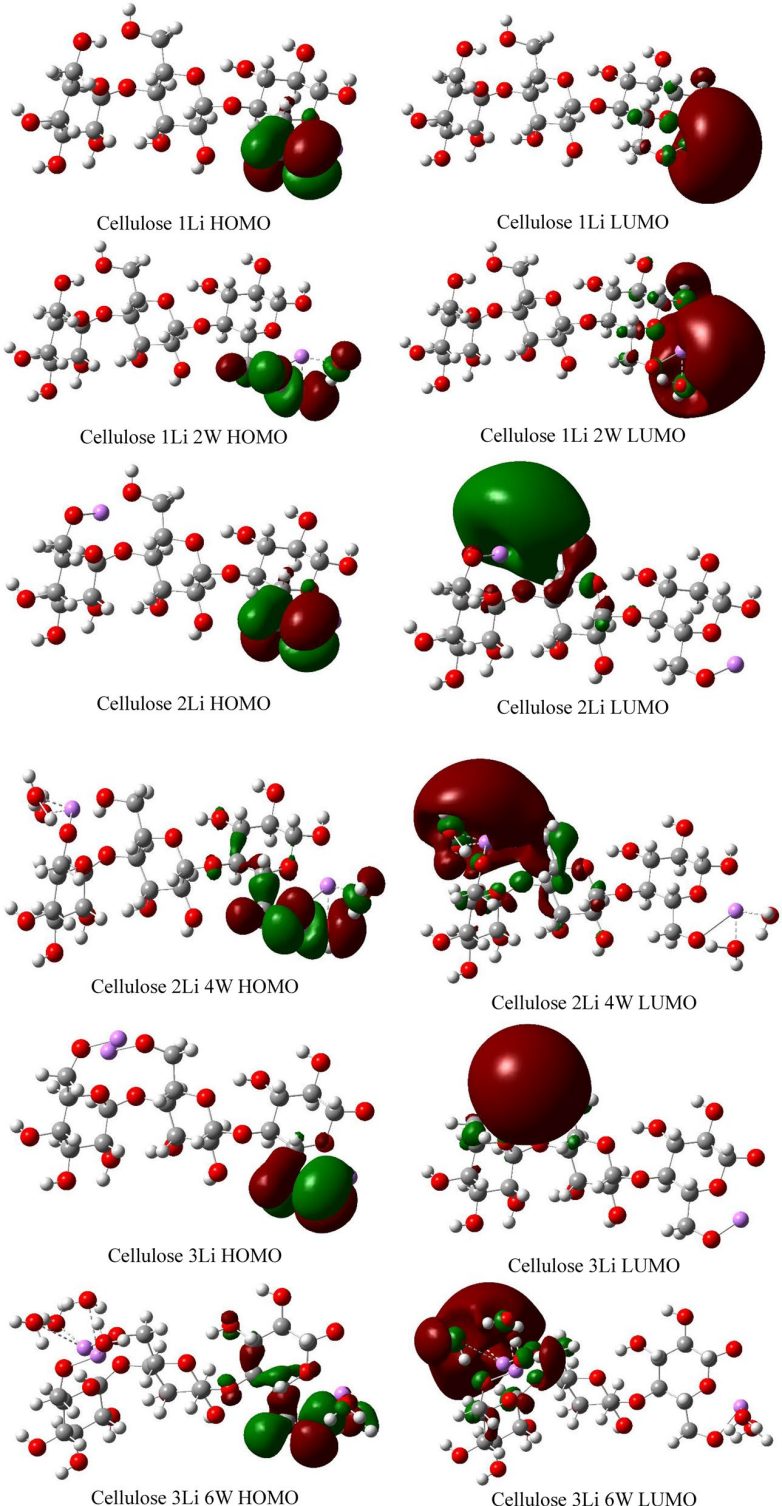
DFT:B3LYP/6-31g(d,p) calculated HOMO/LUMO orbitals of Cellulose-Li.

**Figure 13:**
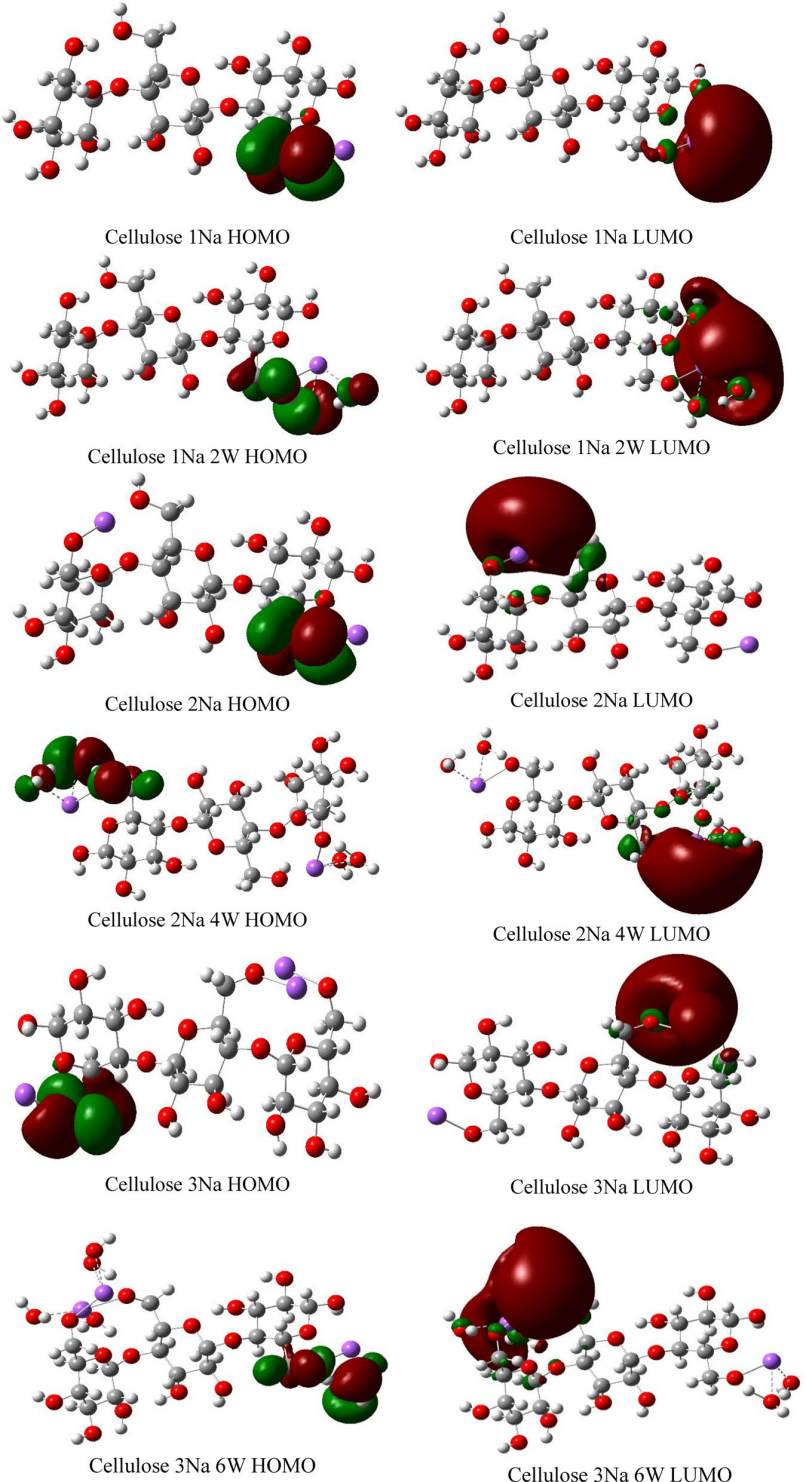
DFT:B3LYP/6-31g(d,p) calculated HOMO/LUMO orbitals of Cellulose-Na.

**Figure 14:**
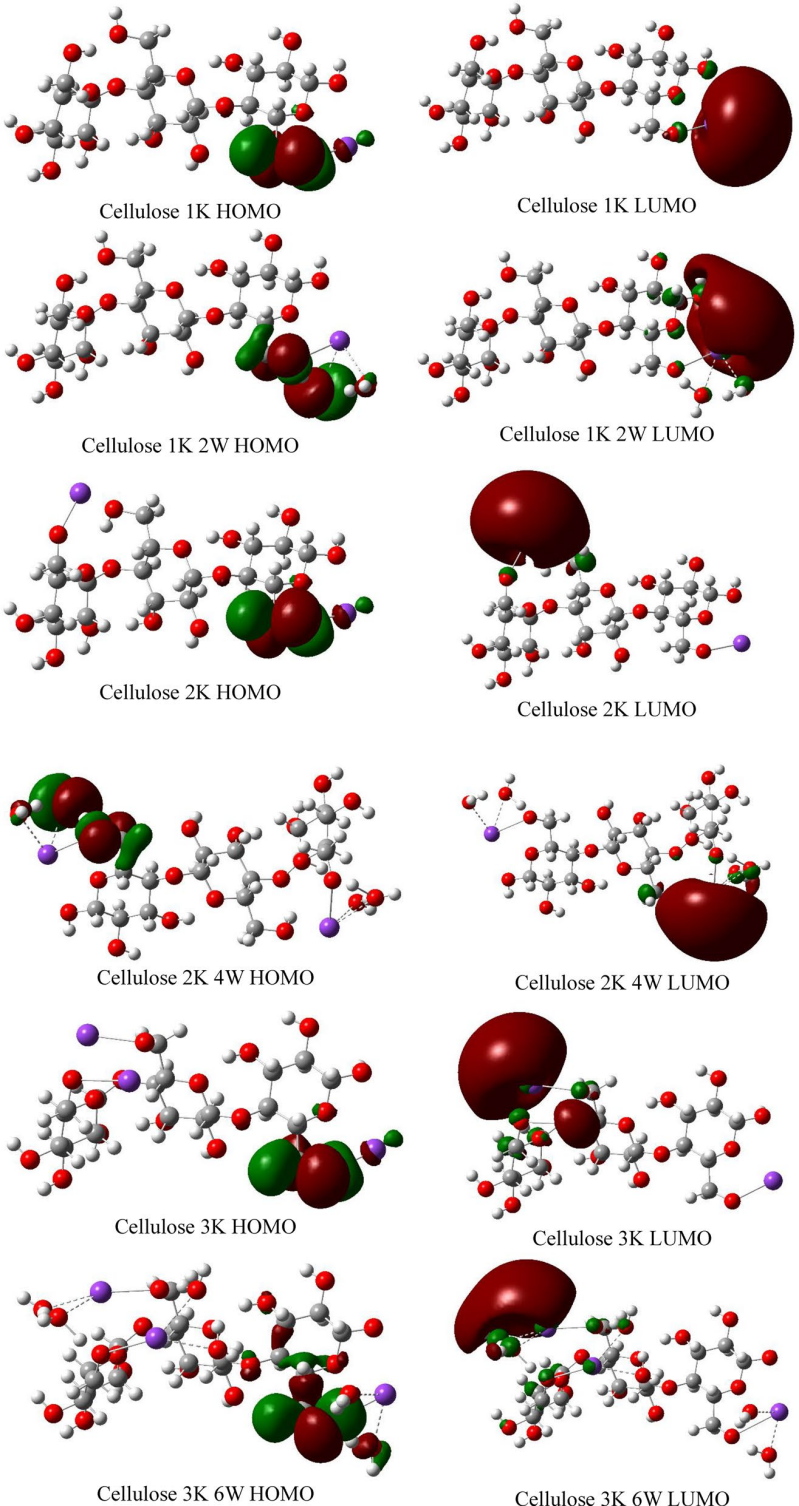
DFT:B3LYP/6-31g(d,p) calculated HOMO/LUMO orbitals of Cellulose-K.

Figures 12, 13 and 14 demonstrate the HOMO–LUMO molecular orbitals distribution of cellulose-Li, cellulose-Na and cellulose-K interactions, respectively. It is clear that the interaction between cellulose and alkali metals resulted in redistribution of both HOMO and LUMO molecular orbitals to be located around the alkali metals both with and without hydration, which means that they increased the reactivity of cellulose.

### Projected density of states

**Figure 15:**
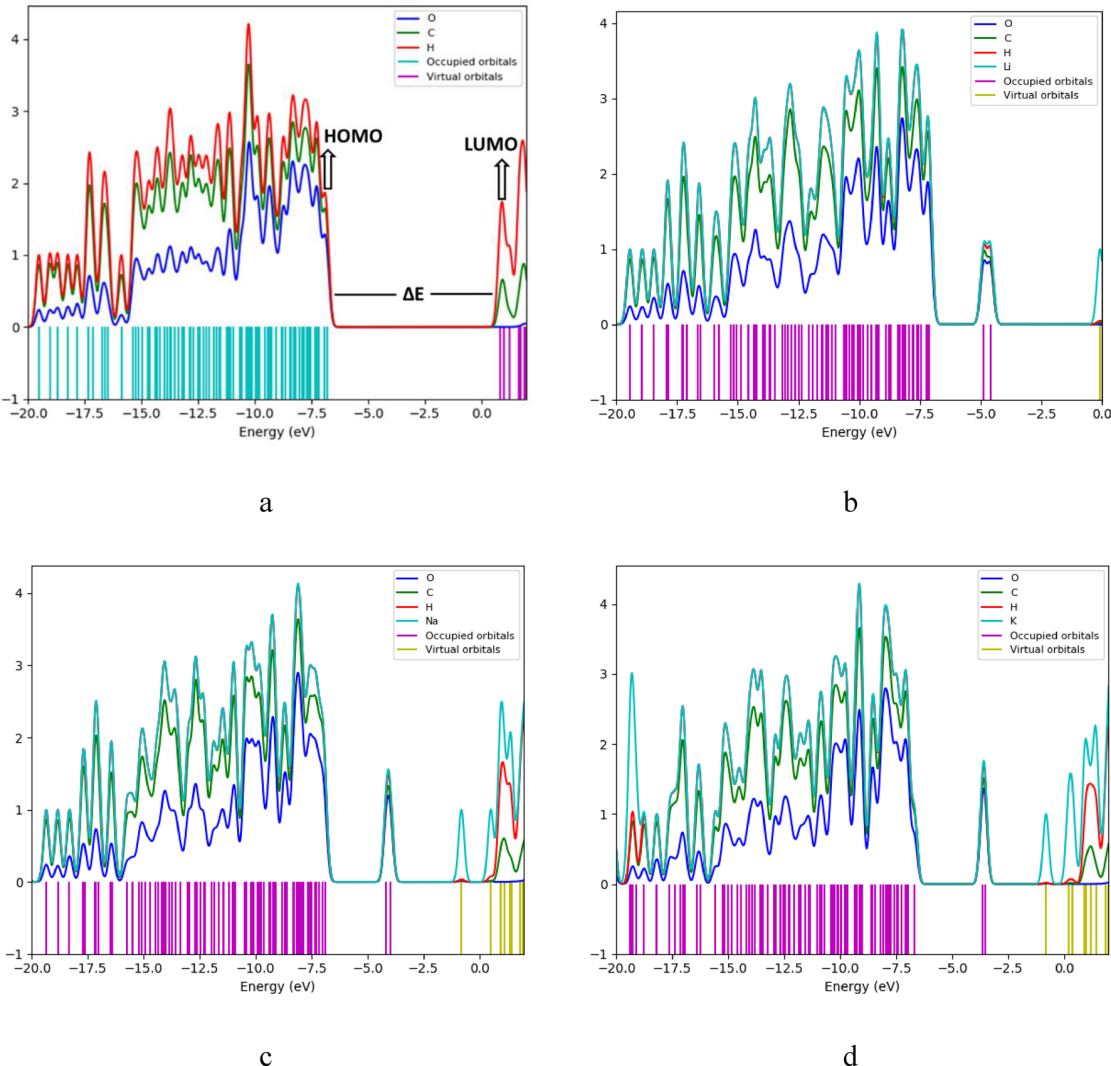
PDOS plots of (**a**) Cellulose; (**b**) Cellulose-1Li; (**c**) Cellulose-1Na and (**d**) Cellulose-1K.

The PDOS plots were also generated in order to reveal the effect of the alkali metals on the electronic structure of cellulose and their contribution in the molecular orbitals of cellulose-Li, cellulose-Na, and cellulose-K interactions. Figure 15 depicts the PDOS plots of cellulose, cellulose-1Li, cellulose-1Na and cellulose-1K. As shown in Fig. 15a, the atomic orbitals of H demonstrated higher contribution for the HOMO than C and O, and this contribution significantly increased more in the LUMO, where the atomic orbitals of O demonstrated no contribution.

In Fig. 15b, the atomic orbitals of O, H, C and Li contributed to the HOMO with Li representing the highest contribution, followed by H, then C, and finally O. The highest contribution for the LUMO was also from the atomic orbitals of Li followed by the atomic orbitals of H, then C, while O demonstrated no contribution.

As shown in Figs. 13d and 15c which depict the cellulose-1Na and cellulose-1K respectively, both interactions demonstrated a similar behavior to that of cellulose-1Li, such that the atomic orbitals of Na and K demonstrated the highest contribution to the molecular orbitals of both HOMO and LUMO. In the PDOS plots of all of the remaining computed interactions (data not shown here) the same behavior was also noticed, where the contribution of the atomic orbitals of the alkali metals was the highest for both HOMO and LUMO both with and without hydration. These results in correlation with the HOMO–LUMO orbital distribution results confirm that the interaction of cellulose with alkali metals both with and without hydration increased the reactivity of cellulose. The rest of the PDOS plots Supplementary Figs. S7–S9.

## Conclusion

DFT B3LYP/6-31G(d,p) molecular modeling calculations were conducted to investigate the spectroscopic and electronic properties of cellulose. Cellulose had TDM of 9.106 Debye and ΔE of 7.647 eV. TDM decreased for all of the proposed interactions, but increased with hydration, while ΔE decreased in all interactions, confirming that cellulose-alkali metal interactions, especially with hydration, resulted in more reactive structures. Mapping both HOMO–LUMO and MESP indicated significant change in the electron density distribution around cellulose under the effect of interaction with the alkali metals, both with and without hydration.

DFT-computed Raman shifts demonstrated a similar behavior to that of computed IR, in which a noticeable difference between the Raman shifts of cellulose, and cellulose-Li, cellulose-Na and cellulose-K was detected in the Raman peaks of the stretching vibrations of OH groups of cellulose. The lower-wavenumber OH peak (3626 cm^−1^) shifted towards higher wavenumbers in cellulose-Li, cellulose-Na and cellulose-K, owing to the change in the O–H chemical bond upon replacement of the H atom with the alkali metal atom with the consequent changes in bond force and distance, as well as charge density. The higher-wavenumber OH peak (3669 cm^−1^) demonstrated significant decrease in its intensity owing to the consumption of OH group with the replacement of H atom by alkali metal atom. These are in a good agreement with the previous findings. The theoretical calculations were experimentally verified using FTIR and FT-Raman spectroscopy, and they showed comparable results.

Finally, the plots of PDOS also clearly demonstrated the contribution of each alkali metal as well as water in the molecular orbitals, reflecting their effect on the electronic properties of cellulose and cellulose-alkali metals composites.

### Supplementary Information

Supplementary Figures.

## Data Availability

The data will be available upon request. Contact Ahmed Refaat: am.refaat@nrc.sci.eg; ahmed_refaat21@yahoo.com.
