# Supplementary material for: Effect of alkali metals on physical and spectroscopic properties of cellulose
Source: Sci Rep. 2023 Dec 8;13:21649. doi: 10.1038/s41598-023-48850-7 (PMC10709645; doi:10.1038/s41598-023-48850-7)
Supplement: Supplementary file 1 — Supplementary Figures. [file 41598_2023_48850_MOESM1_ESM.docx]

**Supplementary Figures**

| 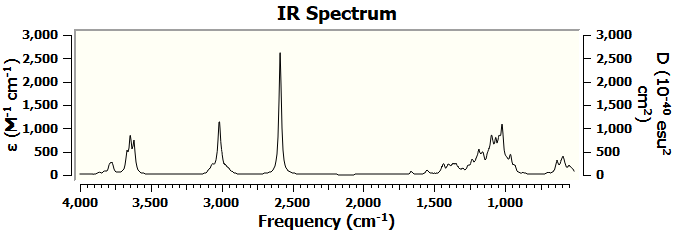  a |
| --- |
| 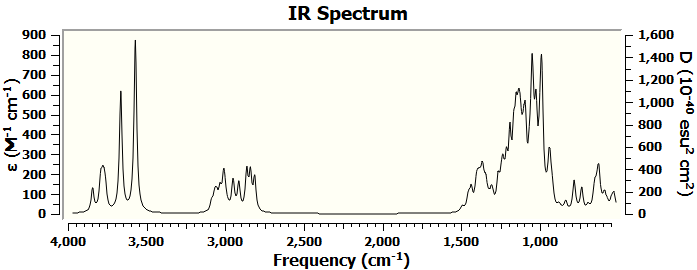  b |
| 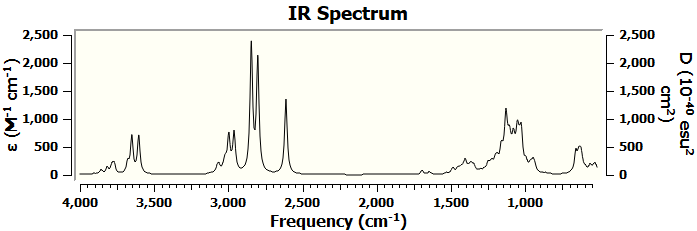  c |
| 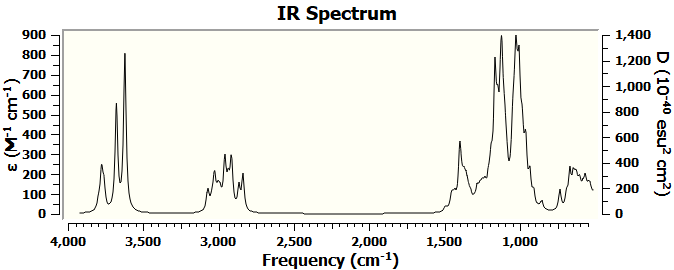  d |
| 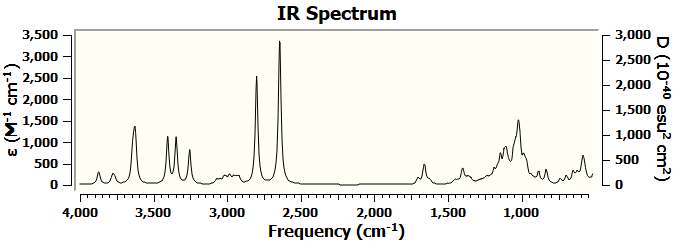  e |

**Supplementary Fig. S1.** DFT:B3LYP/6-31g(d,p) computed IR spectra of a- Cellulose-1Li2W; b- Cellulose-2Li; c- Cellulose-2Li4W; d- Cellulose-3Li and e- Cellulose-3Li6W

| 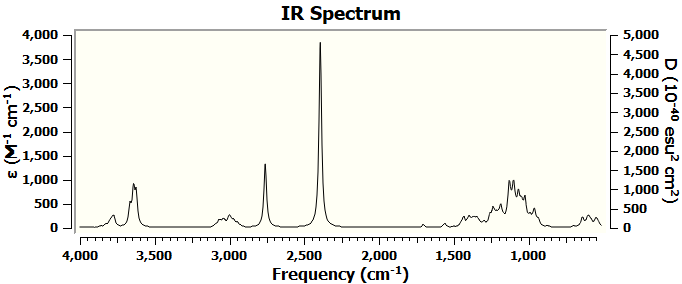  a |
| --- |
| 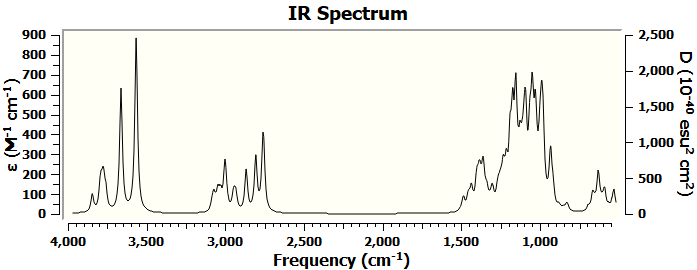  b |
| 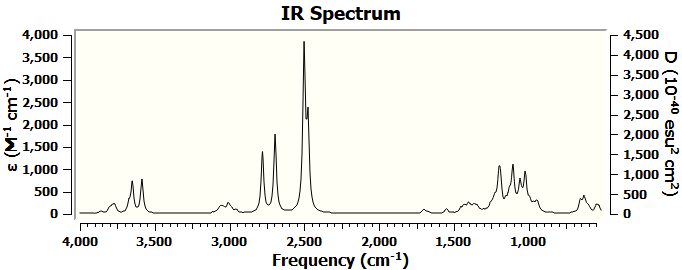  c |
| 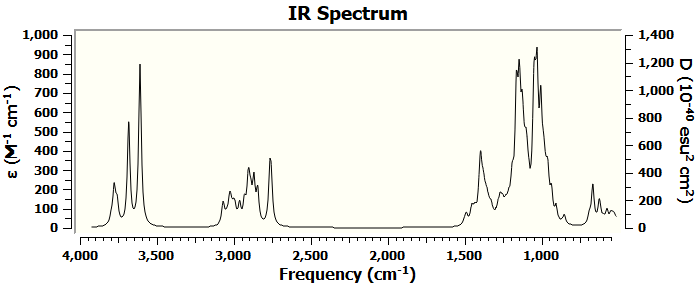  d |
| 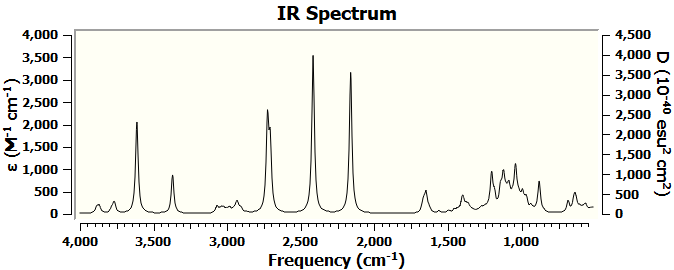  e |

**Supplementary Fig. S2.** DFT:B3LYP/6-31g(d,p) computed IR spectra of a- Cellulose-1Na2W; b- Cellulose-2Na; c- Cellulose-2Na4W; d- Cellulose-3Na and e- Cellulose-3Na6W

| 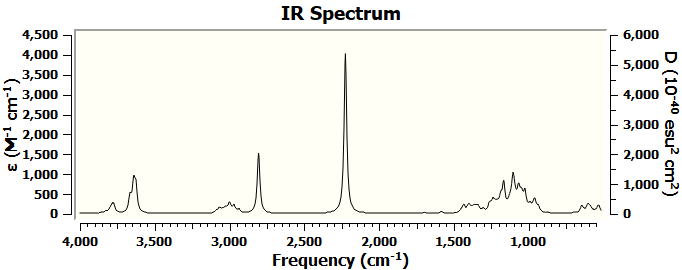  a |
| --- |
| 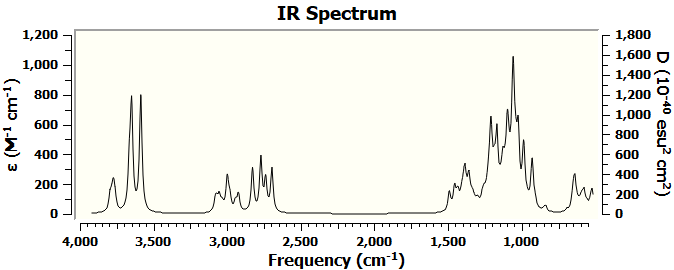  b |
| 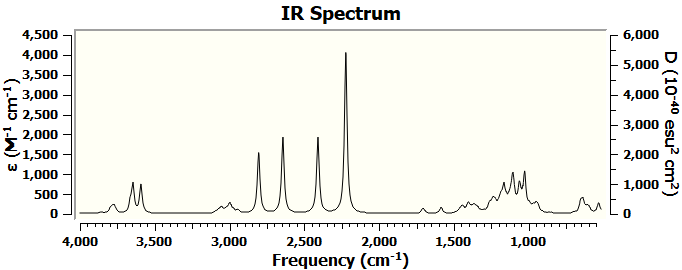  c |
| 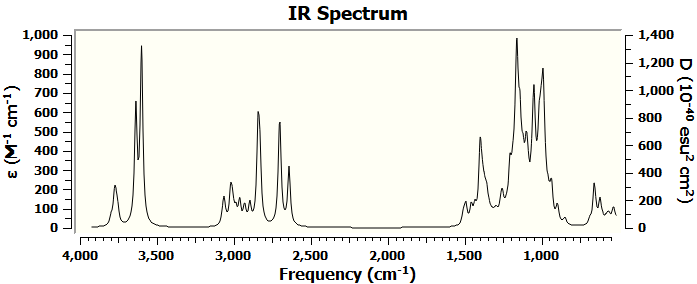  d |
| 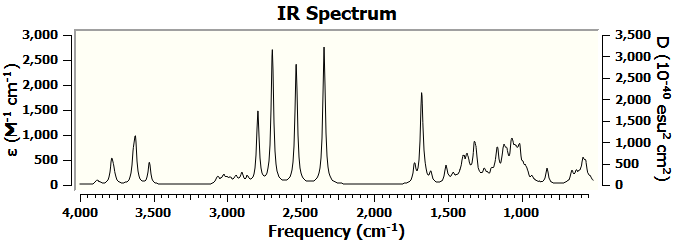  e |

**Supplementary Fig. S3.** DFT:B3LYP/6-31g(d,p) computed IR spectra of a- Cellulose-1K2W; b- Cellulose-2K; c- Cellulose-2K4W; d- Cellulose-3K and e- Cellulose-3K6W

| 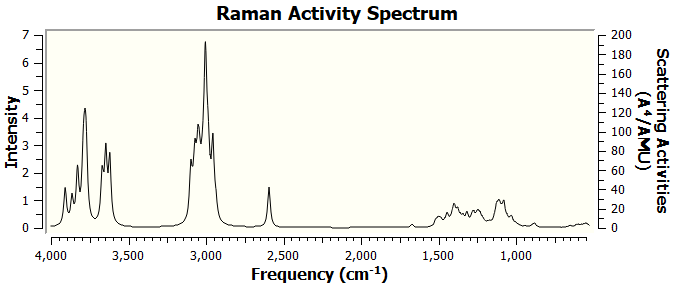  a |
| --- |
| 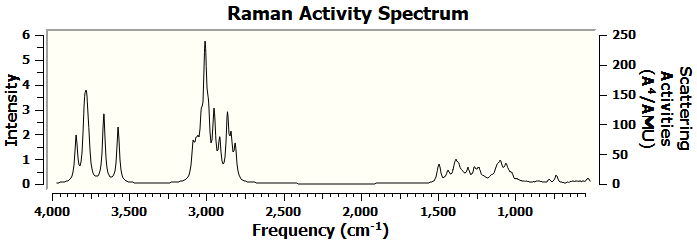  b |
| 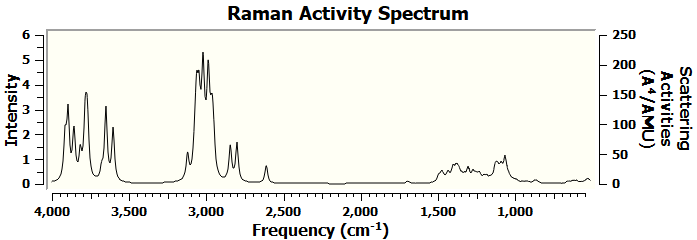  c |
| 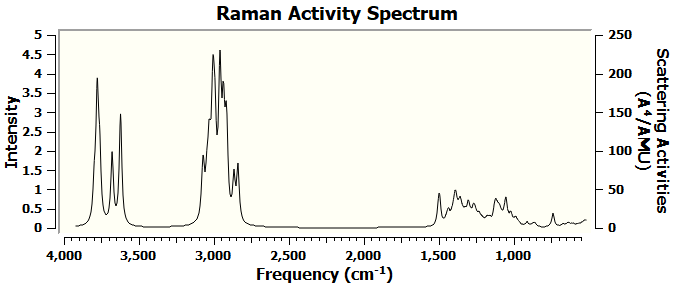  d |
| 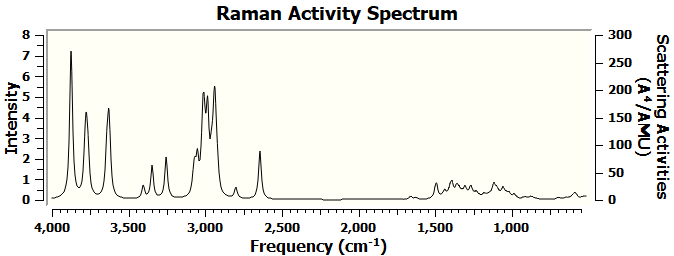  e |

**Supplementary Fig. S4.** DFT:B3LYP/6-31g(d,p) computed Raman shifts of a- Cellulose-1Li2W; b- Cellulose-2Li; c- Cellulose-2Li4W; d- Cellulose-3Li and e- Cellulose-3Li6W

| 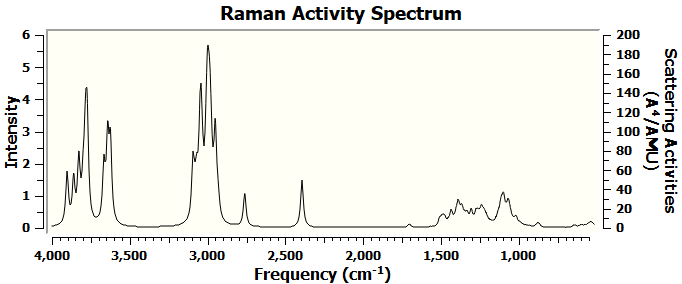  a |
| --- |
| 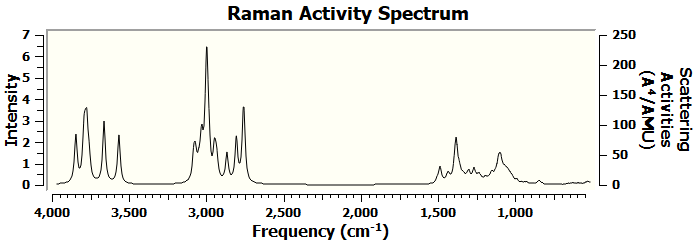  b |
| 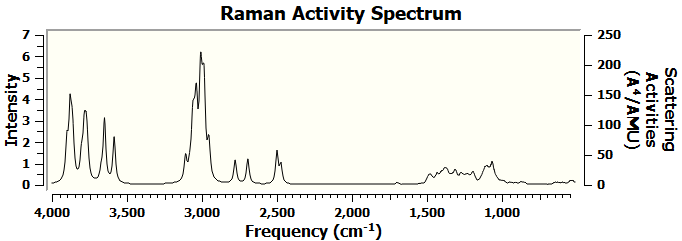  c |
| 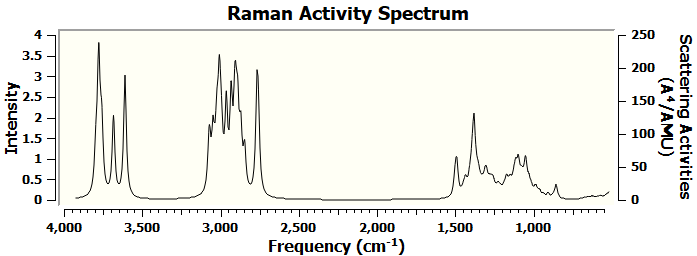  d |
| 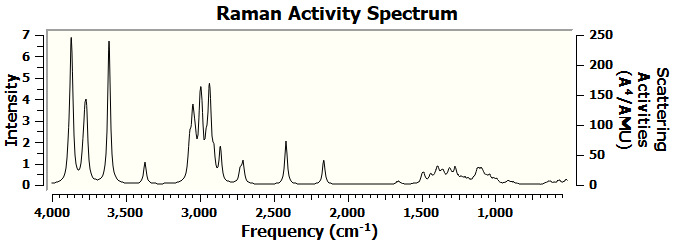  e |

**Supplementary Fig. S5.** DFT:B3LYP/6-31g(d,p) computed Raman shifts of a- Cellulose-1Na2W; b- Cellulose-2Na; c- Cellulose-2Na4W; d- Cellulose-3Na and e- Cellulose-3Na6W

| 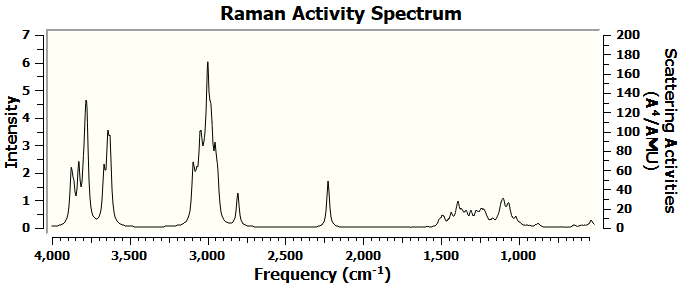  a |
| --- |
| 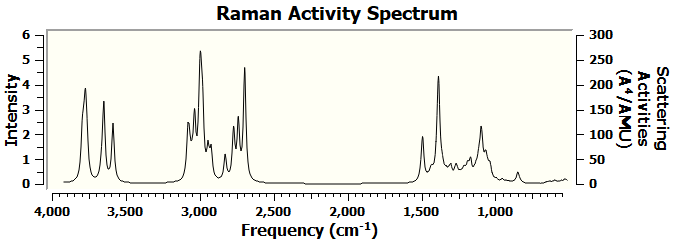  b |
| 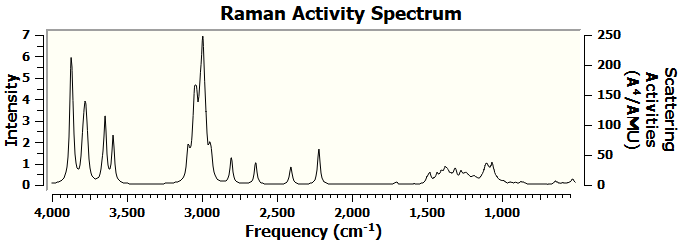  c |
| 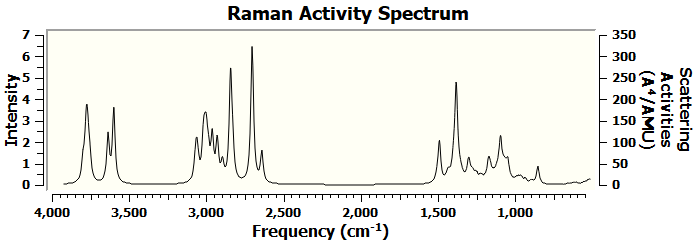  d |
| 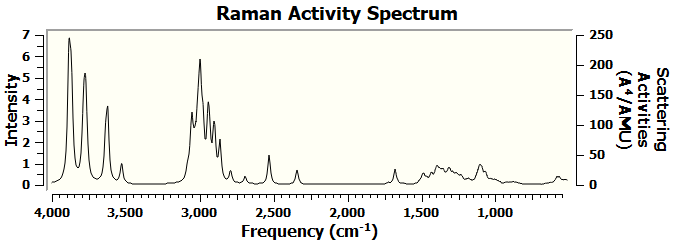  e |

**Supplementary Fig. S6.** DFT:B3LYP/6-31g(d,p) computed Raman shifts of a- Cellulose-1K2W; b- Cellulose-2K; c- Cellulose-2K4W; d- Cellulose-3K and e- Cellulose-3K6W

| 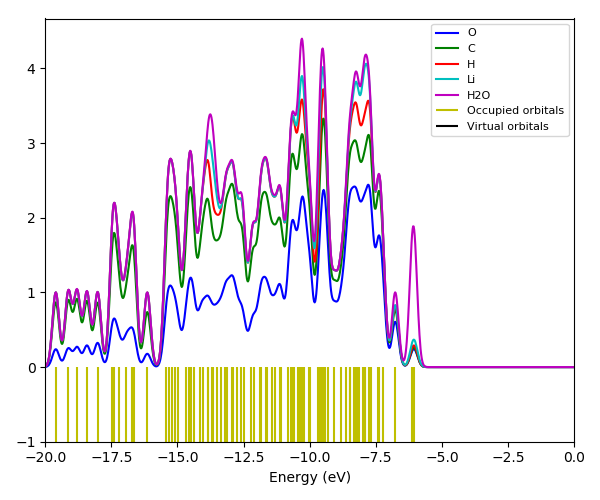  a | 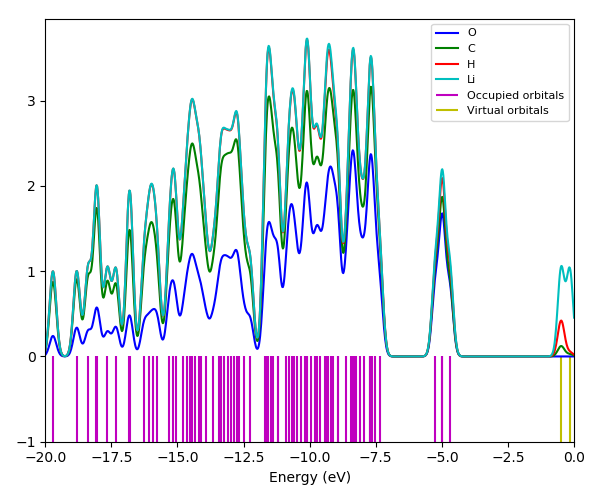  b |
| --- | --- |
| 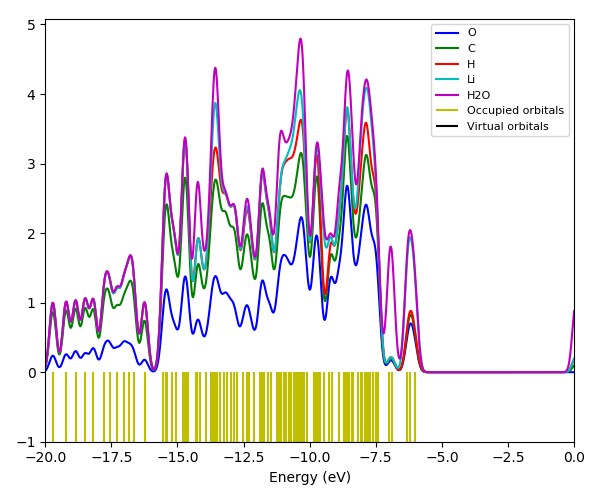  c | 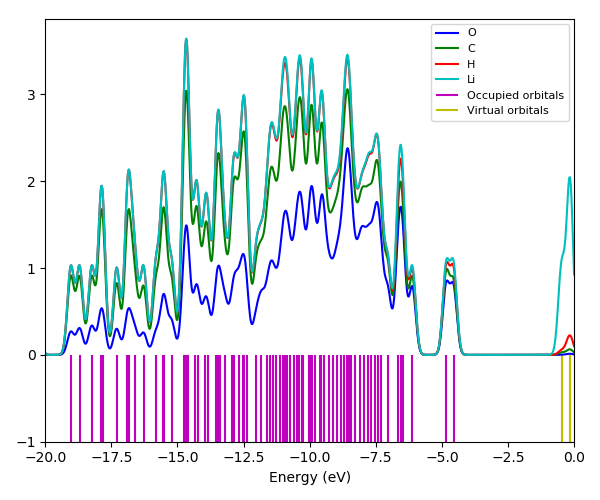  d |
| 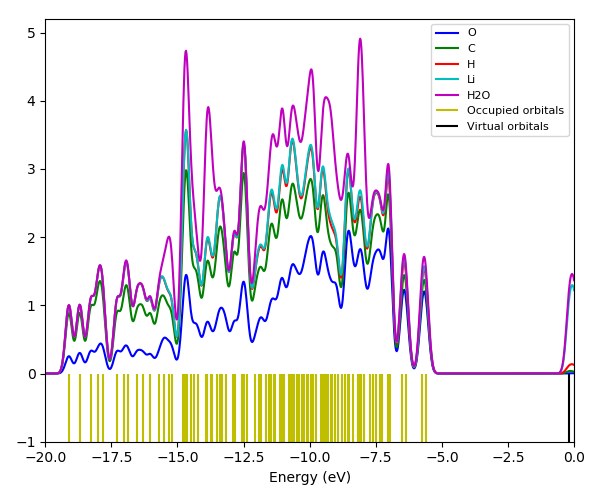  e | |

**Supplementary Fig. S7.** PDOS plots of a- Cellulose-1Li2W; b- Cellulose-2Li; c- Cellulose-2Li4W; d- Cellulose-3Li and e- Cellulose-3Li6W

| 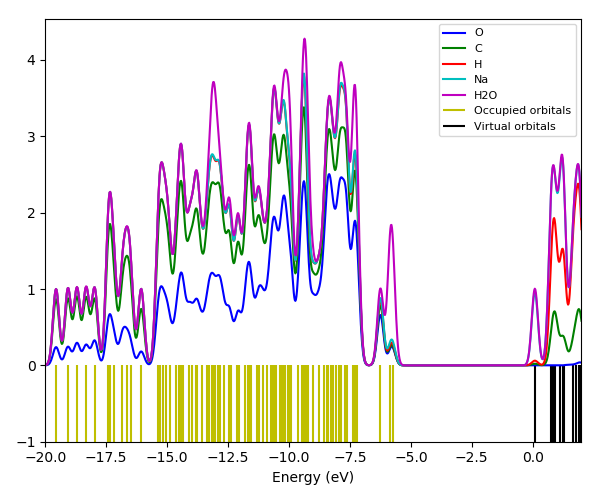  a | 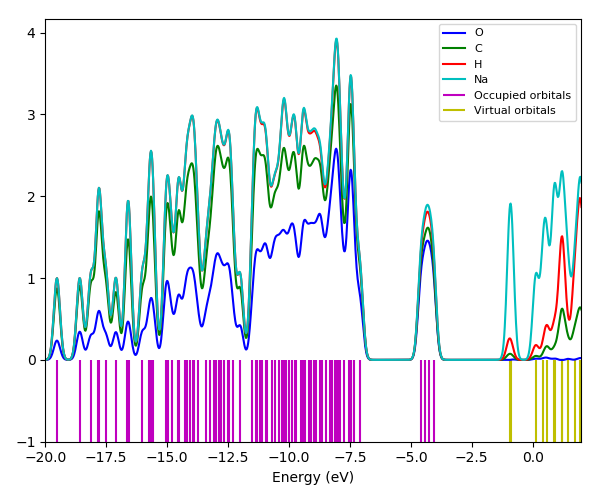  b |
| --- | --- |
| 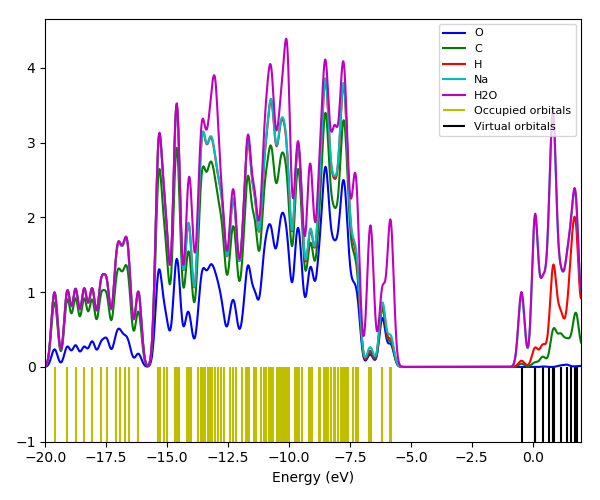  c | 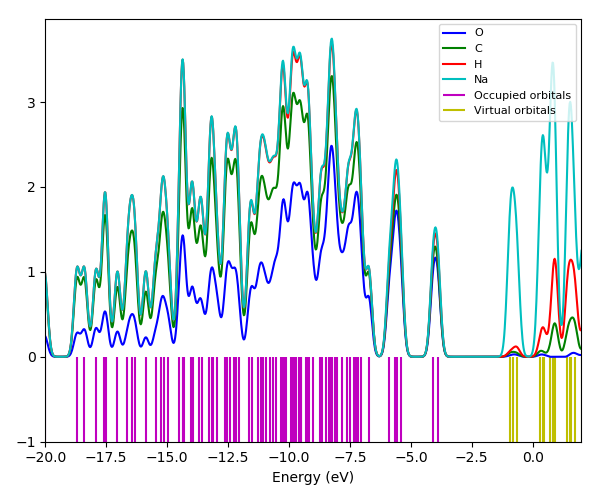  d |
| 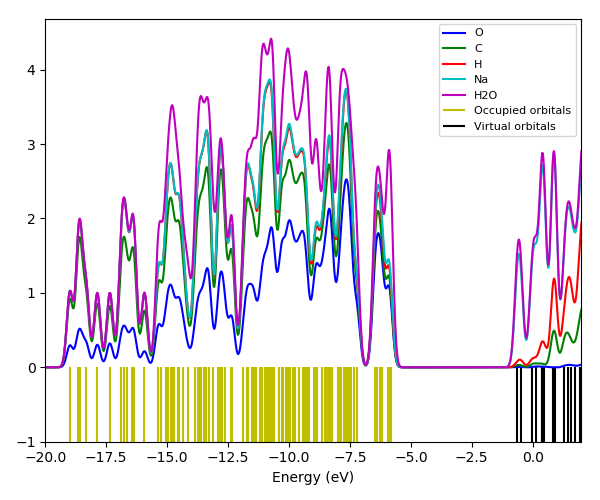  e | |

**Supplementary Fig. S8.** PDOS plots of a- Cellulose-1Na2W; b- Cellulose-2Na; c- Cellulose-2Na4W; d- Cellulose-3Na and e- Cellulose-3Na6W

| 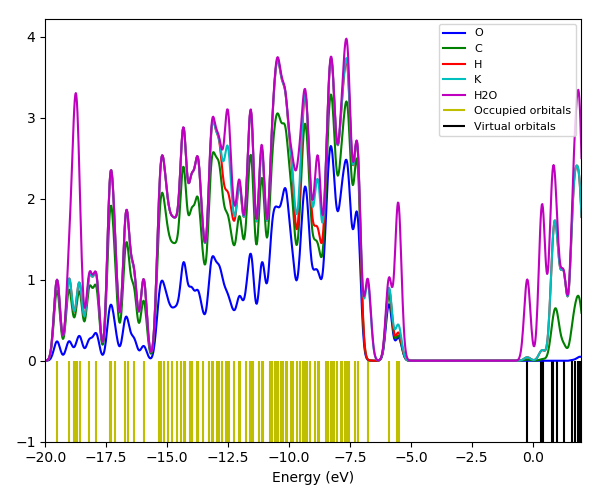  a | 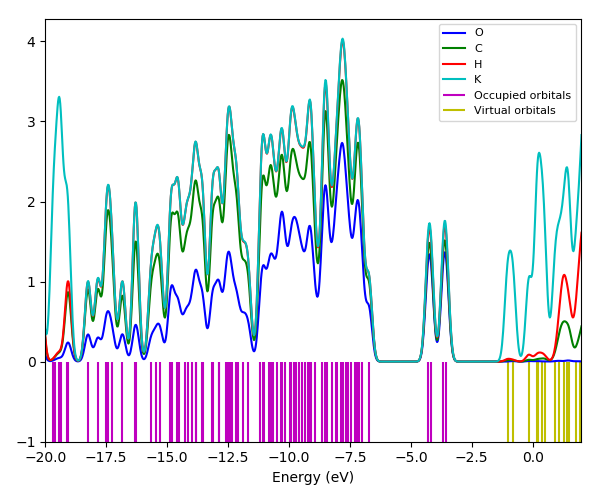  b |
| --- | --- |
| 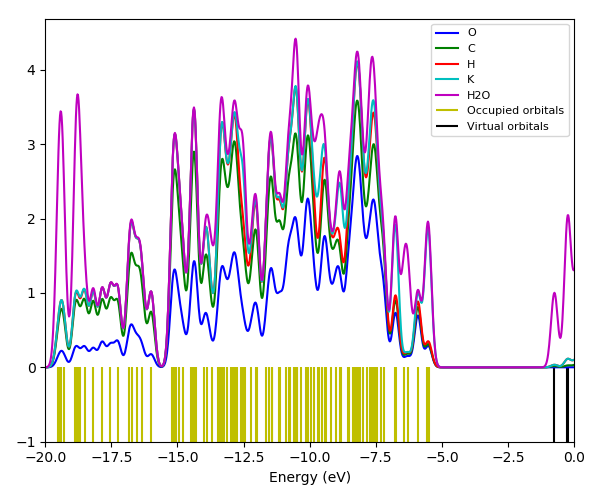  c | 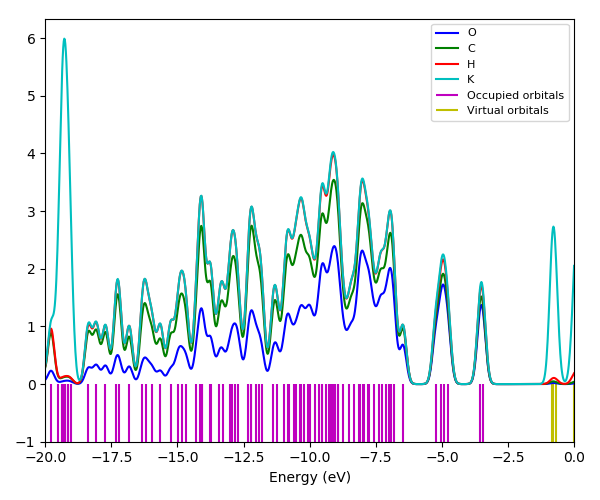  d |
| 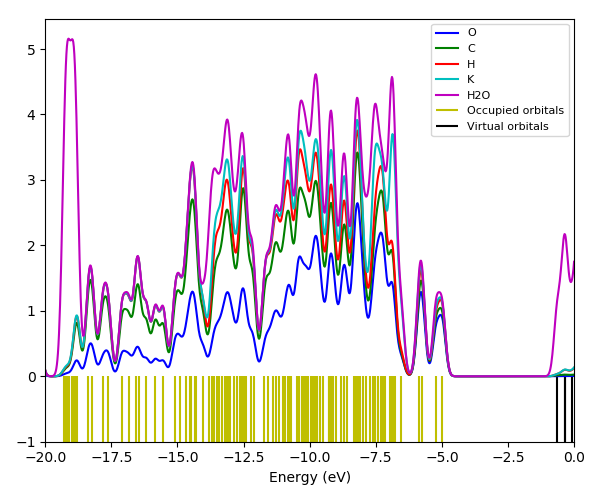  e | |

**Supplementary Fig. S9.** PDOS plots of a- Cellulose-1K2W; b- Cellulose-2K; c- Cellulose-2K4W; d- Cellulose-3K and e- Cellulose-3K6W
